# Lineage-specific duplication of amphioxus retinoic acid degrading enzymes (CYP26) resulted in sub-functionalization of patterning and homeostatic roles

**DOI:** 10.1186/s12862-016-0863-1

**Published:** 2017-01-19

**Authors:** João E. Carvalho, Maria Theodosiou, Jie Chen, Pascale Chevret, Susana Alvarez, Angel R. De Lera, Vincent Laudet, Jenifer C. Croce, Michael Schubert

**Affiliations:** 1 Sorbonne Universités, UPMC Université Paris 06, CNRS, Laboratoire de Biologie du Développement de Villefranche-sur-Mer, Observatoire Océanologique de Villefranche-sur-Mer, 181 Chemin du Lazaret, 06230 Villefranche-sur-Mer, France; 2 Molecular Zoology Team, Institut de Génomique Fonctionnelle de Lyon, Université de Lyon, Université Lyon 1, CNRS, INRA, Ecole Normale Supérieure de Lyon, 46 Allée d’Italie, 69364 Lyon, Cedex 07 France; 3 Laboratoire de Biométrie et Biologie Evolutive, Université de Lyon, Université Lyon 1, CNRS, 43 Boulevard du 11 novembre 1918, 69622 Villeurbanne, France; 4 Departamento de Química Organica, Facultad de Química, Universidade de Vigo, 36310 Vigo, Spain; 5 Present Address: Key Laboratory of Freshwater Aquatic Genetic Resources, Shanghai Ocean University, Huchenghuan Road 999, Shanghai, 201306 China; 6 Present Address: Observatoire Océanologique de Banyuls-sur-Mer, UMR CNRS 7232, Université Pierre et Marie Curie Paris, 1 avenue du Fontaulé, 66650 Banyuls-sur-Mer, France

**Keywords:** *Branchiostoma lanceolatum*, Cephalochordate, Duplication-Degeneration-Complementation (DDC) model, Lancelet, Retinoic acid (RA) signaling

## Abstract

**Background:**

During embryogenesis, tight regulation of retinoic acid (RA) availability is fundamental for normal development. In parallel to RA synthesis, a negative feedback loop controlled by RA catabolizing enzymes of the cytochrome P450 subfamily 26 (CYP26) is crucial. In vertebrates, the functions of the three CYP26 enzymes (CYP26A1, CYP26B1, and CYP26C1) have been well characterized. By contrast, outside vertebrates, little is known about CYP26 complements and their biological roles. In an effort to characterize the evolutionary diversification of RA catabolism, we studied the *CYP26* genes of the cephalochordate amphioxus (*Branchiostoma lanceolatum*), a basal chordate with a vertebrate-like genome that has not undergone the massive, large-scale duplications of vertebrates.

**Results:**

In the present study, we found that amphioxus also possess three *CYP26* genes (*CYP26-1*, *CYP26*-2, and *CYP26-3*) that are clustered in the genome and originated by lineage-specific duplication. The amphioxus *CYP26* cluster thus represents a useful model to assess adaptive evolutionary changes of the RA signaling system following gene duplication. The characterization of amphioxus *CYP26* expression, function, and regulation by RA signaling demonstrated that, despite the independent origins of *CYP26* duplicates in amphioxus and vertebrates, they convergently assume two main roles during development: RA-dependent patterning and protection against fluctuations of RA levels. Our analysis suggested that in amphioxus RA-dependent patterning is sustained by CYP26-2, while RA homeostasis is mediated by CYP26-1 and CYP26-3. Furthermore, comparisons of the regulatory regions of *CYP26* genes of different bilaterian animals indicated that a CYP26-driven negative feedback system was present in the last common ancestor of deuterostomes, but not in that of bilaterians.

**Conclusions:**

Altogether, this work reveals the evolutionary origins of the RA-dependent regulation of *CYP26* genes and highlights convergent functions for CYP26 enzymes that originated by independent duplication events, hence establishing a novel selective mechanism for the genomic retention of gene duplicates.

**Electronic supplementary material:**

The online version of this article (doi:10.1186/s12862-016-0863-1) contains supplementary material, which is available to authorized users.

## Background

During animal development, the vitamin A-derived morphogen retinoic acid (RA) mediates a number of crucial functions, including, for example, early embryonic patterning and organogenesis, by acting on different cellular processes ranging from proliferation to cell death [1–7]. In vertebrates, normal development requires a very tightly controlled balance of the total amount of available RA, which is maintained through positive and negative feedback loops associated, respectively, with RA production (chiefly by RALDH1, 2, and 3, for retinaldehyde dehydrogenase 1, 2, and 3) and RA degradation (chiefly by CYP26A1, B1, and C1, for cytochrome P450 subfamily 26A1, B1, and C1) [8–12]. The biological response to endogenous RA, in turn, is mediated by heterodimers of two nuclear receptors, the retinoic acid receptor (RAR) and the retinoid X receptor (RXR), with the expression levels of RAR in particular being tightly linked to the availability of RA [1, 3, 4]. RAR/RXR heterodimers directly exert their transcriptional function by binding to RA response elements (RAREs) in the regulatory regions of RA target genes [13]. A typical RARE is composed of two direct repeats (DRs) corresponding to a conserved nucleotide sequence [(A/G)G(G/T)TCA] separated by a spacer composed of one, two or five nucleotides (corresponding to, respectively, DR1, DR2, and DR5 elements) [13–15]. Upon RA binding, RAR/RXR heterodimers generally function as ligand-activated transcription factors, but can also mediate RA-dependent repression of target genes in a context-specific manner, the exact molecular modalities of which still remain to be established [16].

During vertebrate development, the RA degrading enzymes of the CYP26 subfamily play critical roles in the formation of an anterior-posterior (A-P) RA gradient as well as in the compensation of RA level fluctuations by oxidizing RA into biologically inactive compounds [17]. They are thus characterized by dynamic, yet highly specific, developmental expression patterns in vertebrates [18], with *CYP26A1*, for example, being expressed in the anterior ectoderm in the early embryo and subsequently becoming localized, amongst other tissues, to the hindbrain, the pharyngeal arches, and the tail bud. Similarly, both *CYP26B1* and *CYP26C1* are detectable in specific rhombomeres of the hindbrain and in pharyngeal arches as well as in fin and limb buds of the developing embryo [18]. Concomitantly, the loss of CYP26 function has been associated both with A-P patterning defects, most prominently in the developing central nervous system (CNS) and the mesoderm, and an increased sensitivity to RA teratogenicity [19]. For instance, *CYP26A1* knockout mice are characterized by a posteriorization of the hindbrain and the vertebral column, and *CYP26B1* genetic ablation leads to craniofacial and limb malformations [20]. Interestingly, while the loss of *CYP26C1* alone does not result in overt anatomical abnormalities [21], the combined removal of *CYP26C1* with either *CYP26A1* or *CYP26B1* induces phenotypes that are more severe than those aforementioned, thereby suggesting that *CYP26C1* plays an important cooperative role in the CYP26-mediated control of endogenous RA levels during vertebrate development [19, 21].

In line with this cooperative action of CYP26 enzymes, the vertebrate RA signaling system in general is characterized by complex feedback mechanisms that are mediated, either indirectly or directly, by RAR/RXR-dependent signaling. As an example of an indirect regulation, it has been shown that, in the vertebrate trunk, RA, generated by RALDH activity, represses and confines *FGF8* expression to rostral and caudal domains (i.e. to the heart- and tail bud-associated progenitor fields) [16]. This action is mediated by RAR/RXR heterodimers binding to a repressive DR2 RARE located upstream of the *FGF8* gene [22, 23] that, in turn, activates *CYP26* expression both anteriorly and posteriorly to limit the extent of RA activity [16, 24, 25]. In addition, the expression of *CYP26A1* and *CYP26B1* have been shown to be dependent on RA activity, thereby generating a *CYP26*-controlled negative feedback loop in RA sensitive tissues to reduce the overall amount of available RA [9, 18, 26, 27]. For *CYP26A1*, this regulation is directly mediated by RAR/RXR heterodimers binding to DR5 RAREs in the promoter region, while for *CYP26B1* this control seems to be indirect [26, 27]. Note further that the vertebrate *CYP26C1* gene is likely to contribute differently than its paralogs to this negative feedback system, as *CYP26C1* expression is actually downregulated following RA stimulation [9].

The intricate molecular mechanisms controlling the catabolism of endogenous RA during vertebrate development likely arose at the base of this lineage following the whole genome duplication (WGD) events that took place during early vertebrate diversification [28, 29]. Therefore, the evolutionary elaboration of the RA signaling system in general seems to be tightly linked to the duplication of RA metabolism genes. The so-called DDC model (for Duplication-Degeneration-Complementation) predicts three possible outcomes following duplication of a gene: non-functionalization (i.e. the loss of one of the duplicates), neo-functionalization (i.e. one of the copies retains the ancestral role, while the other duplicate assumes a novel functionality) or sub-functionalization (i.e. both duplicates assume a part of the function of the single ancestral gene) [30, 31]. While the model predicts that the most likely outcome following duplication of a gene is the loss of one of the duplicates (i.e. non-functionalization), very clear examples for the neo-functionalization and the sub-functionalization of duplicated genes remain scarce [32, 33].

In order to develop a credible scenario for the evolutionary diversification of the vertebrate RA system and investigate the implications of the DDC model in the duplication of RA metabolism genes, we decided to study the function and regulation of RA degradation during embryonic development of the cephalochordate amphioxus (*Branchiostoma lanceolatum*). Due to its phylogenetic position at the base of chordates, amphioxus is a very useful model to characterize chordate- and vertebrate-specific innovations, both on a morphological and a genomic level. For instance, at the morphological level, amphioxus and vertebrates share a dorsal CNS, a postanal tail as well as pharyngeal gill slits [34, 35], while, conversely, amphioxus lacks some vertebrate-specific characters, such as definitive neural crest and placodes as well as a cartilaginous or bony skeleton [34, 35]. Furthermore, amphioxus is a basal chordate that did not undergo WGD [36, 37] and that possesses a vertebrate-like RA signaling pathway [29, 36]. Thus, while RA signaling in vertebrates is generally controlled by three RARs (RARα, RARβ, RARγ) and three RXRs (RXRα, RXRβ, RXRγ) that form a multitude of different heterodimers, the amphioxus genome contains only one *RAR* and one *RXR* gene [38]. Nevertheless, administration of exogenous RA during amphioxus gastrulation leads, as observed in vertebrates, to the posteriorization of the amphioxus CNS and endoderm, hence preventing, for example, the formation of mouth and gill slits [38–43]. These regionalization defects are further associated with a deregulation of *RAR* and *Hox* gene expression, which have been shown to be direct targets of RA signaling in amphioxus, as they are in vertebrates [44].

In amphioxus, three *CYP26* genes (*CYP26-1*, *CYP26-2*, and *CYP26-3*) have been reported, which are clustered together in the genome and have possibly emerged from a lineage-specific duplication [29]. This *CYP26* locus offers a rare, if not unique, opportunity to investigate the adaptive changes following lineage-specific duplication that led to the retention of three *CYP26* genes in the genome. The results from our analyses thus show that the three amphioxus *CYP26* genes arose by lineage-specific tandem duplication of a single, ancestral *CYP26* gene. They further provide evidence that these three genes assume two main functions during amphioxus development, as they do in vertebrates, i.e. patterning of the embryo and protection against RA level fluctuations. These two roles have been sub-functionalized in amphioxus with *CYP26-2* mediating RA-dependent developmental patterning and *CYP26-1* and *CYP26-3* assuming the protection of the embryo from RA teratogenesis. Moreover, the presence of functional RAREs in the amphioxus *CYP26* cluster indicates that RA degradation is regulated in cephalochordates like in vertebrates, i.e. directly by RAR/RXR heterodimers, hence establishing a negative RA feedback system. Comparative genomic analyses of *CYP26* regulatory regions from different bilaterian animals further suggest that this CYP26-dependent negative RA feedback system is not unique to chordates, but probably arose earlier in animal evolution and was already present in the last common ancestor of all deuterostomes, but not in that of all bilaterians. The adaptive advantages of an elaborate CYP26-driven RA degradation system are discussed. In sum, the evolutionary history of amphioxus *CYP26* genes provides an excellent example for the sub-functionalization of two distinct developmental functions and a paradigm for understanding the selective mechanisms acting on duplicated genes and leading to their retention in the genome.

## Results

### *CYP26* genes were duplicated independently several times in bilaterian evolution

Previous analyses have reported three *CYP26* genes in the Florida amphioxus, *Branchiostoma floridae*, and have suggested that they likely originated by lineage-specific duplication from a single ancestral *CYP26* gene [29, 36]. Here, we have identified and cloned three *CYP26* genes from the European amphioxus, *Branchiostoma lanceolatum*. To further assess the phylogenetic relationships of the amphioxus *CYP26* genes relative to each other and to other members of the *CYP26* subfamily, thereby distinguishing between orthologous and paralogous *CYP26* genes, we first carried out phylogenetic analyses using as outgroup the *CYP51* genes, which constitute the *CYP* subfamily that is most closely related to the *CYP2*6 genes [45]. For this phylogenetic tree reconstruction, we used all *CYP26* sequences from 15 vertebrates and 13 invertebrates, including three cephalochordate species (*B. lanceolatum*, *B. floridae*, and *B. belcheri*). Of note, while we successfully identified genes encoding CYP26 in the genomes of priapulids, brachiopods, mollusks, annelids, sea urchins, hemichordates, cephalochordates, ascidian tunicates, and vertebrates, we were unable to do so in those of nematodes and arthropods (as previously reported [2, 46]).

Within vertebrates, we found three *CYP26* paralogs in both cyclostomes (*CYP26A1*, *CYP26B1/C1a*, and *CYP26B1/C1b*) and gnathostomes (*CYP26A1*, *CYP26B1*, and *CYP26C1*). Multiple *CYP26* paralogs were also identified in most invertebrates species studied (two in *Capitella teleta*, *Ciona intestinalis*, *Lottia gigantea, Priapulus caudatus*, and *Saccoglossus kowalevskii*, three in *B. lanceolatum*, *B. floridae*, *B. belcheri*, *Crassostrea gigas*, and *Ptychodera flava*, and four in *Lingula anatina*), with the notable exceptions of the cephalopod *Octopus bimaculoides* and the sea urchin *Strongylocentrotus purpuratus*, each of which possesses only a single *CYP26* gene (Additional file 1).

**Fig. 1:**
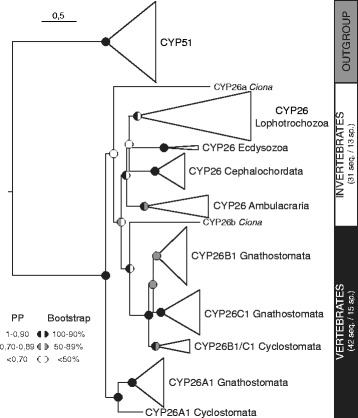
Phylogenetic analysis of the *CYP26* subfamily. Diagrammatic summary of Bayesian Inference (BI) and Maximum Likelihood (ML) analyses of the phylogenetic relationships within the *CYP26* subfamily, with *CYP51* used as outgroup. The detailed tree is shown in Additional file 2 and sequence information is given in Additional file 1. Branch lengths are representative of the amino acid substitution rate and branch support is indicated at each major node as posterior probabilities (PP) for the BI tree and as bootstrap percentages for the ML tree. Furthermore, the total number of sequences (seq.) and species (sp.) is provided for the *CYP26* subfamily

The results of the phylogenetic analysis (Fig. 1 and Additional file 2), obtained with both the Bayesian Inference (BI) and the Maximum Likelihood (ML) methods, suggested an early phylogenetic separation of the vertebrate *CYP26A1* sequences from the vertebrate *CYP26B1/C1* sequences. Vertebrate *CYP26A1* and *CYP26B1/C1* thus formed two independent clades within the *CYP26* subfamily, both of which being strongly supported: 0,91/96 (posterior probability/bootstrap percentage) for *CYP26A1* and 1/99 for *CYP26B1/C1*. Within these two vertebrate *CYP26* clades, the cyclostome sequences consistently branched at the base: *Lethenteron japonicum CYP26A1* at the base of the vertebrate *CYP26A1* (0,96/98) and *CYP26B1/C1a* and *CYP26B1/C1b* from *L. japonicum* and *Petromyzon marinus* at the base of the vertebrate *CYP26B1/C1* (0,7/58). Within the vertebrate *CYP26B1/C1* clade, the association of the gnathostome *CYP26B1* sequences (0,73/58) was less robustly supported than that of the gnathostome *CYP26C1* sequences (1/95), which might be related to the presence of chondrichthyan-specific *CYP26B1* duplicates (*CYP26B1* and *CYP26B2* from *Callorhinchus milii* and *Leucoraja erinacea*) disrupting the base of the *CYP26B1* branch. Of note, while our analysis revealed the general presence of *CYP26A1*, *CYP26B1*, and *CYP26C1* paralogs in chondrichthyans, we were unable to identify a *CYP26A1* gene in *C. milii* and a *CYP26C1* gene in *L. erinacea*. Altogether, these data suggest that the diversification of vertebrate *CYP26* genes was a highly complex process, involving WGD, lineage-specific duplications as well as secondary gene losses.

Outside vertebrates, the *CYP26* sequences from ecdysozoans, ambulacrarians, and cephalochordates always grouped together with very strong support values: the ecdysozoan *P. caudatus* (1/100), the ambulacrarians *P. flava* and *S. kowalevskii* (1/99), and the cephalochordates *B. lanceolatum*, *B. floridae* and *B. belcheri* (1/99). Collectively, these results suggest that, within each of these invertebrate groups, the *CYP26* gene complement originated independently by linage-specific duplication. In contrast, the two sequences from the tunicate *C. intestinalis* did neither associate with each other, nor reliably with one of the major *CYP26* clades in the tree. It is therefore impossible to comment on the nature and origin of the *CYP26* duplication in this animal. Similarly, the reconstruction of the evolutionary history of lophotrochozoan *CYP26* genes is complicated by the lack of phylogenetic resolution between the sequences from the five analyzed lophotrochozoan species, which formed an unresolved polytomy in our analysis. Nonetheless, there is evidence for lineage-specific duplications of *CYP26* genes in the brachiopod *L. anatina*, which possesses four *CYP26* paralogs that established two distinct clades in the tree, one very strongly (1/100) and one very weakly (0,8/--) supported. Furthermore, two of the three *CYP26* sequences from the oyster *C. gigas* are grouped within in a single clade, but the support for this association is very weak (0,95/--). Future studies will thus have to address the processes underlying the evolution of lophotrochozoan *CYP26* genes.

To gain further insights into the diversification of *CYP26* genes in different animal lineages, we next conducted a phylogenetic dating analysis (Additional file 3). This survey indicated that the *CYP26* genes of the ecdysozan *P. caudatus* were likely duplicated independently at the end of the Ordovician (about 446 Mya). Similarly, within the ambulacrarians, we found evidence for linage-specific duplications in hemichordates, which likely occurred during two different periods: the early Carboniferous for *S. kowalevskii* (about 342 Mya) and the middle Triassic for *P. flava* (about 240 Mya). Finally, in chordate lineages, *CYP26* genes have also likely been duplicated independently in cephalochordates, tunicates, and vertebrates, with the three cephalochordate genes resulting from an initial duplication in the early Carboniferous (about 358 Mya), followed by a subsequent duplication during the middle Permian (about 295 Mya). In contrast, while it is difficult to conclude on the timing of the duplication giving rise to the two *CYP26* genes in the ascidian *C. intestinalis*, the evolution of the vertebrate *CYP26* complement is complex and implies a series of duplications, including an ancient split into *CYP26A1* and *CYP26B1/C1* and the subsequent diversification of *CYP26A1*, *CYP26B1*, and *CYP26C1* during the Cambrian period (about 548 to 510 Mya). It should be added that, consistent with the results of the phylogenetic tree, the dating analysis did not yield reliable information on the timing of the duplications of lophotrochozoan *CYP26* genes.

### *CYP26-2* expression is suggestive of a function in developmental patterning of the *B. lanceolatum* embryo

**Fig. 2:**
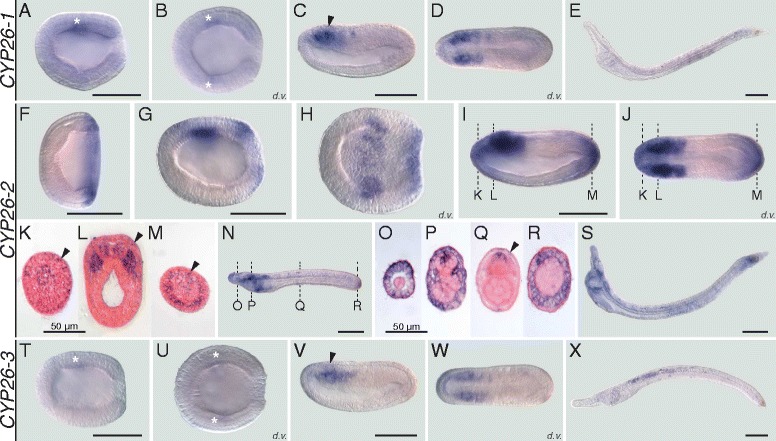
Developmental expression patterns of amphioxus *CYP26* genes. Whole mount in situ hybridization experiments were carried out for *CYP26-1* (**a-e**), *CYP26-2* (**f-s**), and *CYP26-3* (**t-x**). Amphioxus (*Branchiostoma lanceolatum*) embryos and larvae are shown as lateral views with anterior to the left and the dorsal side up, excepting for (**b, d, h, j, u, w**), which are dorsal views (d.v.) with anterior to the left, and for (**k-m**) and (**o-r**), which are cross-sections viewed from the front. Cross-sections in (**k-m**) are through the embryo shown in (**i, j**) at the levels indicated by the dashed lines and cross-sections in (**o-r**) are through the embryo shown in (**n**) at the levels indicated by the dashed lines. *White* asterisks in (**a, b, t, u**) highlight inconspicuous early expression domains of *CYP26-1* and *CYP26-3*. Arrowheads in (**c, v**) indicate central nervous system expression and in (**k-m, q**) ectodermal signal. Developmental stages shown are: early gastrula (**f**), late gastrula (**a, b, g, h, t, u**), mid neurula (**c, d, i-m, v, w**), very early larva (**n-r**), early (60 h) larva (**e, s, x**). Scale bars are 100 μm for the whole mounts and 50 μm for the cross-sections

In vertebrates, *CYP26A1, CYP26B1*, and *CYP26C1* have very distinct expression patterns with several key domains being conserved between different species [6, 18]. Thus, we next assessed the temporal and spatial distribution of the three cephalochordate-specific duplicates in the amphioxus *B. lanceolatum* by *in situ* hybridization (ISH). The ISH results revealed that the expression profiles of *CYP26-1* and *CYP26-3* are generally quite similar (Fig. 2a-e, t-x). For both genes, no signal was detectable by ISH from fertilization through mid gastrulation. Expression of both *CYP26-1* and *CYP26-3* is first identifiable at late gastrula stages as a weak signal in the lateral anterior mesoderm (Fig. 2a, b, t and u). As development proceeds, this domain becomes associated with the most anterior somites at mid neurula stage (Fig. 2c, d, v and w). At this stage, *CYP26-1* and *CYP26-3* are also discreetly and transiently expressed in the anterior central nervous system (CNS), at the level of the first somite (Fig. 2c and v). Expression of both genes remains very weak and chiefly associated with mesodermal tissues during subsequent developmental stages (Fig. 2e and x): while the *CYP26-1* signal is most evident in central and posterior regions of the larva (Fig. 2e), *CYP26-3* is mainly detectable in central and more anterior larval territories (Fig. 2x).

In contrast to *CYP26-1* and *CYP26-3*, *CYP26-2* has a much more complex developmental expression profile during *B. lanceolatum* embryonic and early larval development. Expression of *CYP26-2* is first detectable by ISH at the mid gastrula stage with the signal being localized globally around the blastopore (Fig. 2f). The blastopore-associated signal subsequently weakens and, at the late gastrula stage, an additional expression domain appears in a region corresponding to presumptive lateral mesoderm and anterior neuroectoderm (Fig. 2g and h). By the mid neurula stage, the mesodermal signal has been expanded into the two anterior-most somite pairs (Fig. 2i, j and l). At this stage, *CYP26-2* is further still detectable in the anterior neuroectodem (Fig. 2i, j and l). Additionally, the gene is now expressed in the ectoderm, most conspicuously in the anterior and posterior tips of the embryo (Fig. 2i, j and k-m), as well as in the anterior- and posterior-most endoderm (Fig. 2i, j and m). At the mid neurula stage, the blastopore-associated signal becomes perceivable in the newly formed tail bud (Fig. 2f-j and m). In very early larvae, just before the opening of the larval mouth, expression of *CYP26-2* is detectable anteriorly in all germ layers, i.e. the ectoderm, the mesoderm, the endoderm as well as in the CNS, with the signal being least noticeable in the endoderm (Fig. 2n-p). Furthermore, in both ectoderm and CNS, individual cells are labeled along the A-P body axis (Fig. 2n and q) and, at the posterior end of the embryo, the tail ectoderm strongly expresses *CYP26-2* (Fig. 2n and r). In the amphioxus larva, the overall domains of *CYP26-2* expression are maintained, with conspicuous labeling anteriorly and posteriorly and a weaker signal in the center (Fig. 2s). In sum, while *CYP26-1* and *CYP26-3* expression is very discreet and chiefly limited to the mesoderm, that of *CYP26-2* is detectable in all germ layers and dynamically changes in space and time throughout development.

### *CYP26* acts as a fine regulator of RA levels in the patterning of the *B. lanceolatum* larval tail fin

**Fig. 3:**
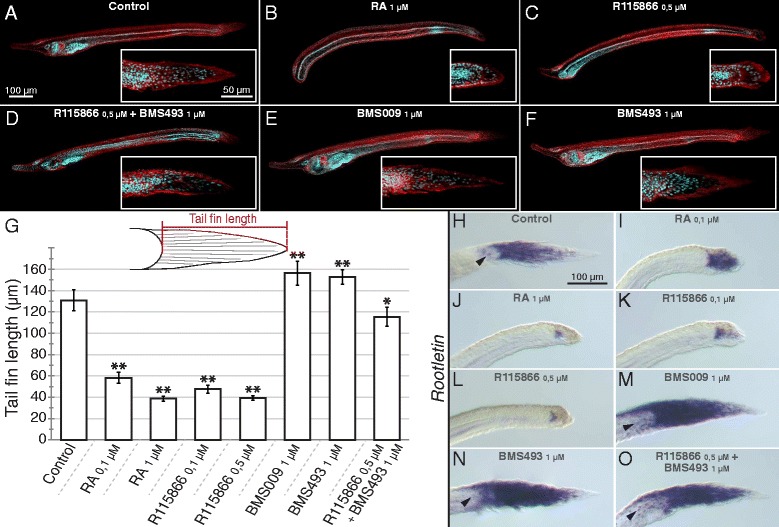
Effects of retinoic acid (RA) signaling alterations on the development of amphioxus. (**a-f**) Early amphioxus (*Branchiostoma lanceolatum*) larvae at 60 h of development with anterior to the left and the dorsal side up are shown as maximal projections of confocal microscopy scans displaying auto-fluorescence (in *red*) and Hoechst nuclear staining (in *cyan*). Magnifications of the tail region are boxed, with anterior to the left and the dorsal side up. Scale bars are 100 μm for the larvae and 50 μm for the magnified tail fins. (**g**) Tail fin length measurements following pharmacological treatments. The length of the tail fin of *B. lanceolatum* larvae at 60 h of development was measured, as indicated in the schematics. The graph shows the tail fin lengths ± standard deviation, with *n* = 15, for the different treatment conditions indicated. Tail fin lengths that are significantly different from the DMSO control are indicated as “*”, for a cutoff value of *p* = 0,005, and as “**”, for a cutoff value of *p* = 0,001. (**h-o**) *Rootletin* expression in the tail fin of early *B. lanceolatum* larvae at 60 h of development, with anterior to the left and the dorsal side up. Arrowheads in (**h, m-o**) indicate expression in lateral ectodermal cells. Pharmacological treatments are as indicated. Scale bars are 100 μm

Disruption of CYP26 activity causes very severe defects during vertebrate development [18]. To determine the role of CYP26 enzymes in the amphioxus embryo, we subsequently disrupted endogenous RA degradation during amphioxus development by treatments with the CYP26-specific inhibitor R115866 [47, 48]. For comparisons, the R115866 treatments were carried out in parallel to treatments with RA or with two different RAR antagonists (BMS009 and BMS493). The capacity of R115866 to inhibit endogenous RA degradation was verified by double treatments of R115866 and BMS493. The results obtained from these different pharmacological treatments of *B. lanceolatum* embryos are in large agreement between each other and with previous studies that characterized the roles of RA signaling in amphioxus endoderm specification and pharyngeal patterning [38–43]. Thus, while the downregulation of RA signaling activity by RAR antagonists (1 μM of either BMS009 or BMS493) resulted in an enlarged pharynx and an expansion of pharyngeal structures (Fig. 3e and f), the upregulation of endogenous RA signaling by 1 μM RA led to a shortening of the pharynx and the malformation of pharyngeal structures (such as the mouth and the gill slits) (Fig. 3b). Consistently, the local upregulation of RA signaling by 0,5 μM R115866 yielded similar results (Fig. 3c), and co-treatments of 0,5 μM R115866 and 1 μM BMS493 led to an attenuation of the severe phenotype induced by 0,5 μM R115866 alone with at least a partial recovery of pharynx formation and patterning (Fig. 3d).

Importantly, these pharmacology-based experiments revealed a previously undescribed role of RA signaling in developmental patterning of the amphioxus larval tail fin, a finding that is consistent with localized expression of *CYP26-2* in the amphioxus tail fin ectoderm. At 60 h of development, amphioxus larvae are characterized by an ectodermal tail fin that is pointy in shape and on average 130,2 μm long (Fig. 3a and g). When amphioxus embryos were treated with RA at the gastrula stage, the resulting tail fins of 60-h larvae are round and significantly shorter. These effects were observable with exogenous treatments of both 0,1 μM and 1 μM RA (Fig. 3b and g). Similar results were obtained with R115866 treatments at 0,1 μM and 0,5 μM (Fig. 3c and g). Conversely, RAR antagonist treatments, with either BMS009 or BMS493, had the inverse effect: the tail fin becomes pointier in shape and is slightly elongated (Fig. 3e-g). Co-treatment of 0,5 μM R115866 with 1 μM BMS493 led to a partial rescue of the tail fin phenotype with an almost normal shape and a slight reduction of the overall length (Fig. 3d and g).

It has previously been shown that the amphioxus larval tail fin is composed of columnar epidermal cells that contain a large ciliary rootlet [49, 50] and that RA signaling promotes tail regression in late, pre-metamorphic *B. floridae* larvae by downregulating the gene encoding the main component of the ciliary rootlet: the protein Rootletin [51]. However, the regulation of *Rootletin* expression by RA signaling in the tail fin of early *B. floridae* larvae has not yet been reported. Given the effects on early tail fin formation we observed in *B. lanceolatum* in response to the alteration of endogenous RA signaling levels, we decided to investigate the patterns of *Rootletin* expression in 60-h *B. lanceolatum* larvae following the pharmacological treatment regimes detailed above. As previously described for late *B. floridae* larvae [51], *Rootletin* is expressed in the basal compartment of the columnar tail fin cells in 60-h *B. lanceolatum* larvae (Fig. 3h). At this developmental stage, the gene is further detectable in a small number of lateral ectodermal cells (Fig. 3h).

While treatment with 0,1 μM RA resulted in a marked reduction of *Rootletin* expression concomitant with an apical compaction of the columnar tail fin cells (Fig. 3i), 1 μM RA very strongly restricted the *Rootletin* expression domain (Fig. 3j). The effect on tail fin development of either 0,1 μM or 0,5 μM of the CYP26 inhibitor R115866 was similar to that of exogenous RA and the R115866-treated larvae were thus generally characterized by a significant reduction of *Rootletin* expression (Fig. 3k and l). The RAR antagonists BMS009 and BMS493 led to an apical expansion of *Rootletin* expression as well as to an increase of the overall length of the tail fin (Fig. 3m and n). Furthermore, the RAR antagonist treatments induced *Rootletin* expression in additional lateral ectodermal cells not directly associated with the tail fin (Fig. 3m and n). Intriguingly, while co-treatments of 0,5 μM R115866 and 1 μM BMS493 restored a shortened tail fin with almost normal shape, expression of *Rootletin* remained expanded into the apical territory of the tail fin and detectable in lateral ectodermal cells (Fig. 3o). Altogether, these observations suggest that the formation of the ectodermal tail fin in *B. lanceolatum* is dependent of RA signaling, with CYP26-dependent degradation playing an important role in fine tuning of endogenous RA signaling levels to ensure proper tail fin outgrowth.

### *CYP26-1* and *CYP26-3* are highly responsive to RA and specifically upregulated to avoid teratogenic effects of RA

**Fig. 4:**
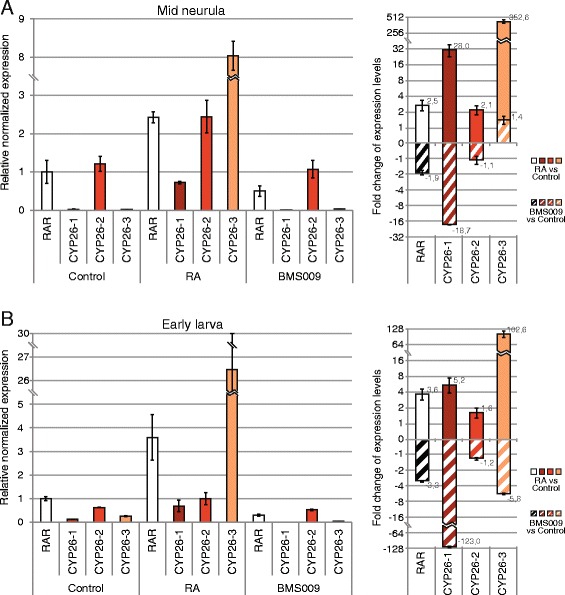
Quantitative changes of amphioxus *CYP26* expression in response to retinoic acid (RA) signaling alterations. Expression of *CYP26-1*, *CYP26-2*, and *CYP26-3* in amphioxus (*Branchiostoma lanceolatum*) was assessed by quantitative real time PCR (qPCR) at two developmental stages (mid neurula at 20 h of development and early larva at 48 h of development). *RAR* expression was also established and used as a normalized positive control. (**a**) Relative normalized expression (*left panel*) and fold change of expression levels (*right panel*) at the mid neurula stage, using a Log_2_ scale. (**b**) Relative normalized expression (*left panel*) and fold change of expression levels (*right panel*) at the early larval stage, using a Log_2_ scale

In vertebrates, CYP26 enzymes are known to function locally to reduce RA levels hence protecting target tissues from RA teratogenesis. In this context, the regulation of CYP26 activity is mediated, at least in part, by RA signaling, which very dynamically up- or down-regulates *CYP26* expression in a tissue-dependent context [18]. To obtain insights into the regulation of *CYP26* genes by RA signaling in *B. lanceolatum*, we performed quantitative real time PCR (qPCR) analyses on amphioxus embryos at two developmental stages (mid neurula and early larva) that have been treated, at the early gastrula stage, with either 1 μM RA or 1 μM of the RAR antagonist BMS009. The qPCR experiments assessed the changes in relative expression of the three *B. lanceolatum CYP26* genes normalized by *RAR* expression in control embryos (Fig. 4a and b).

Through these analyses, we found that the expression levels of *CYP26-1* and *CYP26-3* in control embryos were very low when compared to *CYP26-2* (about 45,0 to 50,0 times less in mid neurulae and 2,5 to 5,0 times less in early larvae) (Fig. 4a and b). RA treatments very significantly increased the expression of both *CYP26-1* and *CYP26-3* at the mid neurula stage (an average of 28 and 352,6 fold, respectively), while *CYP26-2* levels increased merely by about 2,1 fold (Fig. 4a). In contrast, when embryos were treated with the RAR antagonist BMS009, *CYP26-1* levels decreased by an average of 18,7 fold in mid neurulae, while the overall transcription of *CYP26-2* and *CYP26-3* remained relatively unchanged when compared to controls (a decrease of about 1,1 fold for *CYP26-2* and an increase of about 1,4 fold for *CYP26-3*) (Fig. 4a). In early larvae, RA treatment also significantly increased the expression levels of *CYP26-1* and *CYP26-3* (by an average of 5,2 and 102,6 fold, respectively), while that of *CYP26-2* increased only by about 1,6 fold (Fig. 4b). The RAR antagonist BMS009 had the opposite effect and strongly decreased *CYP26-1* levels by about 123,0 fold and *CYP26-3* levels by an average of 5,8 fold (Fig. 4b). In contrast, *CYP26-2* expression dropped by only about 1,2 fold (Fig. 4b). These results indicate that, although *CYP26-*2 is the amphioxus *CYP26* gene most strongly and broadly expressed during embryogenesis, *CYP26-1* and *CYP26-3* are much more reactive to alterations of endogenous RA signaling levels.

**Fig. 5:**
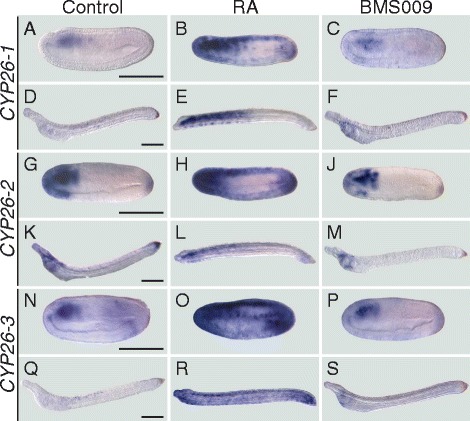
Spatial changes of amphioxus *CYP26* expression in response to retinoic acid (RA) signaling alterations. Whole mount in situ hybridization experiments were carried out for *CYP26-1* (**a-f**), *CYP26-2* (**g-m**), and *CYP26-3* (**n-s**) on amphioxus (*Branchiostoma lanceolatum*) embryos treated at the late blastula stage with either DMSO (as control), all-*trans* RA (1 μM) or RAR antagonist BMS009 (1 μM). The BMS009-treated embryo in (**c**) and larva in (**f**) were subjected to an extended coloration period. Mid neurula embryos at 20 h of development (**a-c, g-j, n-p**) and early larvae at 48 h of development (**d-f, k-m, q-s**) are shown as lateral views with anterior to the left and the dorsal side up. Scale bars are 100 μm

Following this quantification, we next assessed, by ISH, the developmental expression profiles of amphioxus *CYP26* genes upon RA signaling-altering pharmacological treatments. Using the same developmental stages as for the qPCR analyses (mid neurulae and early larvae), we found that the upregulation of *CYP26-1* expression upon RA treatment was not uniform (Fig. 5a-f). At the mid neurula stage, *CYP26-1* was most strongly induced in the anterior half of the embryo in all tissue layers (i.e. in CNS, ectoderm, mesoderm, and endoderm), all along the CNS as well as in the posterior tip of the embryo in all tissue layers excepting the mesoderm (Fig. 5a and b). In early larvae, the gene was upregulated in all tissue layers of the anterior half of the animal, but largely undetectable posteriorly, except in a few cells in the ectoderm (Fig. 5d and e). As expected from the qPCR experiments, expression of *CYP26-1* in embryos and larvae treated with the RAR antagonist BMS009 was very inconspicuous, but, after an extended coloration step, was nonetheless detectable in mesodermal tissues (Fig. 5c and f).

Treatment effects were similar, but not identical, for *CYP26-2*. At the mid neurula stage, RA strongly induced *CYP26-2* anteriorly and posteriorly in all tissue layers as well as all along the CNS and ectoderm (Fig. 5h). However, following RA treatment, *CYP26-2* expression was more conspicuous anteriorly and posteriorly in the embryo, when compared to that of *CYP26-1*. In contrast, in early larvae the effects of RA treatments were less pronounced for *CYP26-2* than for *CYP26-1*. Chiefly, *CYP26-2* expression expanded slightly in the mesoderm and ectoderm in the anterior half of the animal (Fig. 5k and l). Of note, the reduction of *CYP26-2* expression in the anterior endoderm of RA-treated larvae was due to the absence of pharyngeal structures normally expressing the gene [43]. Treatments with the RAR antagonist BMS009 generally weakened the *CYP26-2* signal, most noticeably in the CNS, the mesoderm, and both the anterior and posterior ectoderm (Fig. 5j and m).

Finally, the expression of *CYP26-3* was also very strongly expanded by RA at mid neurula and early larva stages, even more strongly than that of *CYP26-1* with a general expansion of the staining observed throughout the embryo at both stages (Fig. 5n and o). Following RA treatment, expression of the gene was induced anteriorly and posteriorly in all tissue layers, most conspicuously in the center of the embryo in the CNS, ectoderm, and mesoderm (Fig. 5n and o). By the early larval stage, *CYP26-3* remained generally upregulated throughout the animal (Fig. 5q and r), which contrasts with a more restricted distribution of *CYP26-1* and *CYP26-2* transcripts at this stage of development in response to RA treatments (Fig. 5e and l). In embryos and larvae treated with the RAR antagonist BMS009, the *CYP26-3* signal was very weak and chiefly limited to mesodermal tissues (Fig. 5p and s). Altogether, these results show that treatments with RA and RAR antagonist induce similar, but not identical, tissue-specific responses of amphioxus *CYP26-1*, *CYP26-2*, and *CYP26-3*, thereby supporting the notion that each one of the three *CYP26* genes is required for a distinctive set of developmental functions.

### RA regulates *CYP26-1*, *CYP26-2*, and *CYP26-3* directly and via evolutionary conserved functional RAREs present in the *CYP26* cluster

**Fig. 6:**
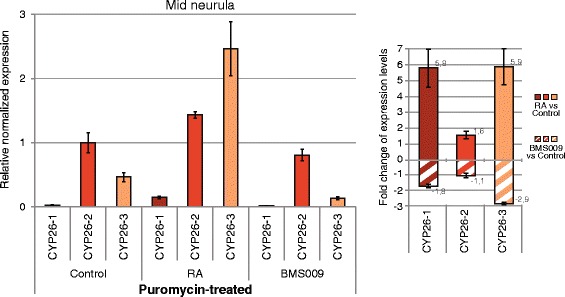
Dynamics of amphioxus *CYP26* gene expression following protein synthesis inhibition and retinoid treatments. Expression of *CYP26-1*, *CYP26-2*, and *CYP26-3* was assessed in amphioxus (*Branchiostoma lanceolatum*) by quantitative real time PCR (qPCR) at the mid neurula stage (at 20 h of development) following protein synthesis inhibition by puromycin treatment (200 μg/ml) at 19 h of development and subsequent treatment with DMSO (as control), all-*trans* RA (1 μM) or RAR antagonist BMS009 (1 μM) for 1 h. Relative normalized expression (*left panel*) and fold change of expression levels (*right panel*) relative to the controls are shown

To assess, whether the observed effects of RA and RAR antagonist on amphioxus *CYP26* expression were mediated directly by RAR/RXR heterodimers, we first carried out pharmacological treatments in the presence of puromycin, a compound that efficiently blocks *de novo* protein synthesis in developing amphioxus [44]. Amphioxus mid neurulae were hence treated with puromycin for 5 min prior to adding RA or the RAR antagonist BMS009. The embryos were then sampled 1 h later and, following RNA extraction, the expression levels of *CYP26-1*, *CYP26-2*, and *CYP26-3* were determined by qPCR (Fig. 6). The results showed that, in the presence of puromycin, RA treatments significantly upregulated expression of both *CYP26-1* and *CYP26-3* (by about 5,8 fold and 5,9 fold, respectively), while that of *CYP26-2* increased more modestly, by about 1,6 fold (Fig. 6). The RAR antagonist BMS009 had the inverse effect, reducing *CYP26-1* and *CYP26-3* levels by about 1,8 fold and 2,9 fold, respectively. In contrast, *CYP26-2* expression stayed relatively stable, decreasing only by about 1,1 fold (Fig. 6). Altogether, these results are consistent with the effects of RA pharmacology on the expression of *CYP26* genes described above. Furthermore, they indicate that the responses to RA and RAR antagonist treatments of all three amphioxus *CYP26* genes do not require *de novo* protein synthesis, thereby implying that they must be mediated directly by RAR/RXR protein heterodimers present in the target tissues.

**Fig. 7:**
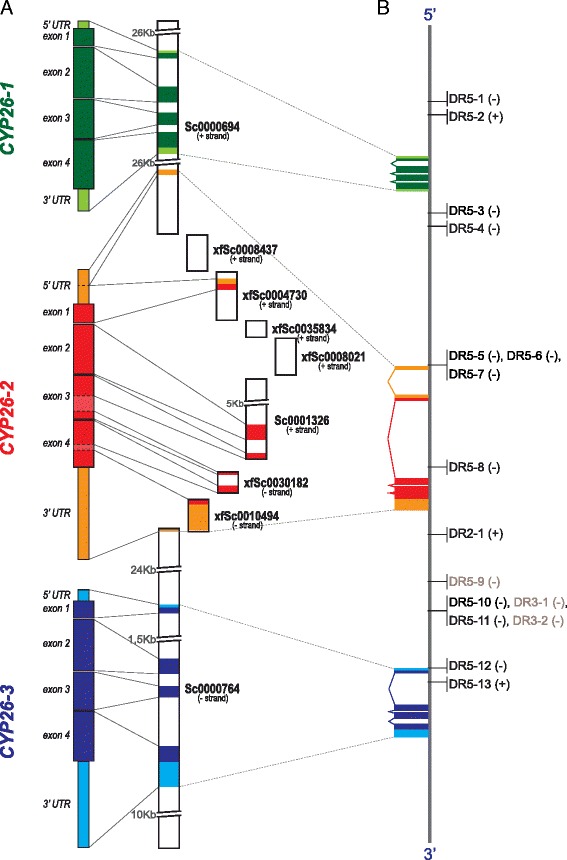
Organization and distribution of conserved retinoic acid response elements (RAREs) in the amphioxus *CYP26* cluster. (**a**) Schematic representation of the experimentally verified amphioxus (*Branchiostoma lanceolatum*) *CYP26-1*, *CYP26-2*, and *CYP26-3* gene sequences, including the 5′ and 3′ untranslated regions (UTRs) and the exons of the coding region, as well as of the corresponding scaffolds of the *B. lanceolatum* genome that cover the complete *CYP26* cluster and thus also include intronic and intergenic regions. The names of the *B. lanceolatum* genome scaffolds are indicated, as is their orientation relative to the *CYP26* cluster (+/− strand). (**b**) Distribution of conserved amphioxus RAREs in the *B. lanceolatum CYP26* cluster. The orientation of each RARE is indicated relative to the *CYP26* cluster (+/−). RAREs recognized and bound by the *B. lanceolatum* RAR/RXR heterodimer in vitro are indicated in *black*, RAREs that do not associate with the *B. lanceolatum* RAR/RXR heterodimer in vitro are shown in *grey*. The RARE sequences are given in Additional file 4 and the in vitro assays are shown in Fig. 8

In order to obtain deeper insights into the mechanisms of the direct regulation of *CYP26-1*, *CYP26-2*, and *CYP26-3* by RA signaling we then investigated the genomic environment of *CYP26* genes in the three amphioxus species with available genomes: *B. floridae* [36, 37], *B. belcheri* [52], and *B. lanceolatum* [53]. Our analyses allowed us to reconstruct the entire genomic clusters for the three amphioxus *CYP26* genes, previously described only for *B. floridae* [29] (Fig. 7). Although variable in size (about 85 Kbp in *B. floridae*, 56 Kbp in *B. belcheri*, and 79 Kbp in *B. lanceolatum* from the start codon of *CYP26-1* to the stop codon of *CYP26-3*), the order of the three *CYP26* genes within the continuous clusters is conserved between the three amphioxus species, lending further support to the notion that these genes originated by tandem duplications from a single ancestral *CYP26* gene at the base of the cephalochordates. Making use of the complete sequences of the three amphioxus *CYP26* clusters, we further searched systematically for conserved RAREs in the vicinity of the *CYP26-1*, *CYP26-2*, and *CYP26-3* genes. This *in silico* survey included a genomic region encompassing 20 Kbp upstream of the *CYP26-1* start codon and 20 Kbp downstream of *CYP26-3* stop codon (Fig. 7). In total, we identified 38 candidate RAREs in *B. floridae,* 30 in *B. belcheri*, and 21 in *B. lanceolatum*. Of these, 16 RAREs were conserved between all three amphioxus species, both in terms of DR motif sequences and their relative position within the *CYP26* cluster (Fig. 7b and Additional file 4).

**Fig. 8:**
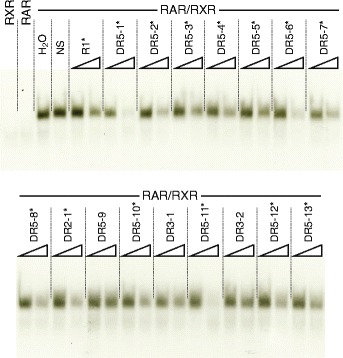
In vitro validation of putative retinoic acid response elements (RAREs) in the amphioxus *CYP26* cluster. Using in vitro synthesized *Branchiostoma lanceolatum* RAR and RXR proteins, the binding of the cognate RAR/RXR heterodimer to putative RAREs identified in the amphioxus *CYP26* cluster was assessed. The mouse *CYP26A1* DR5 sequence (R1) [26] (Table 1) was used as ^32^P-radiolabeled double-stranded probe. The RXR and RAR lanes respectively contained only in vitro synthesized RXR or RAR proteins, together with the radiolabeled probe. The H_2_0 (water) lane contained radiolabeled probe as well as RAR and RXR proteins. The NS (non-specific) lane contained a non-specific and unlabeled double-stranded DR element in 100-fold molar excess relative to the radiolabeled probe as well as RAR and RXR protein. All other lanes contained one of the 16 putative amphioxus DR elements (for nomenclature explanations see Fig. 7b) at 10-fold (*left lane*) or 100-fold (*right lane*) molar excess, along with RAR and RXR protein and the radiolabeled mouse R1 element, to test the binding specificity of the putative amphioxus RAREs. A *black* asterisk indicates that a given DR element can be recognized by the *B. lanceolatum* RAR/RXR heterodimer, as it outcompetes binding to the mouse R1 element

These conserved amphioxus *CYP26* RAREs, i.e. 13 DR5, 1 DR2, and 2 DR3 elements, were subsequently used in EMSA analyses to investigate their capacity, in vitro, to interact with the *B. lanceolatum* RAR/RXR heterodimer. Note that, even though DR3 elements are not considered as common RAREs, they were included in this survey. The results showed that the DR2 element and 12 of the 13 DR5 elements can be recognized and bound by the *B. lanceolatum* RAR/RXR heterodimer (Figs. 7b and 8), indicating that most of these RAREs might be functional in vivo. Furthermore, the consensus signature of the in vitro validated amphioxus DR5 elements [(A/G)G(G/T)T(C/G)A NNN(A/G)(A/C/G) (A/G)G(G/T)(T/A)CA] is very similar to the classical vertebrate DR5 signature [(A/G)G(G/T)TCA (N)_5_ (A/G)G(G/T)TCA] (Additional file 4), suggesting that, as in vertebrates [54, 55], amphioxus *CYP26* genes are regulated directly by RA signaling via RAREs located in close vicinity of the open reading frames (ORFs).

Furthermore, comparisons of the conserved and validated amphioxus *CYP26* RAREs with functional RAREs associated with vertebrate *CYP26* genes revealed that the amphioxus DR5-6 sequence located upstream of *CYP26-2* (Fig. 7b and Additional file 4) is identical in sequence to a DR5 RARE located upstream of vertebrate *CYP26A1* genes [AGTTCA (N)_5_ AGTTCA] [26, 54]. To verify, whether this DR5 RARE motif is a chordate innovation or an ancestral signature of bilaterian *CYP26* genes, we screened the genomic regions surrounding *CYP26* genes in the annelid *C. teleta*, the mollusk *L. gigantea*, the echinoderm *S. purpuratus*, the hemichordate *S. kowalevskii*, and three vertebrates (*Takifugu rubripes*, *Mus musculus*, and *Homo sapiens*)*.* While no DR5 RARE with a similar motif could be identified in the annelid *C. teleta* and the mollusk *L. gigantea*, the conserved DR5 RARE was recovered in all three vertebrate species as well as in the hemichordate *S. kowalevskii*. Intriguingly, in the echinoderm *S. purpuratus*, instead of a DR5 RARE, we found a DR2 RARE with two similar DR sequences [AGTTCA] in an inverse orientation relative to the conserved DR5 RAREs in other species (Table 1). Of note, the conserved amphioxus DR5 RARE is located significantly further away from the *CYP26* start codon than in the other studied species. Although the biological significance of this finding still remains to be explored, these results nonetheless suggest that the direct control of *CYP26* expression by RA signaling, mediated at least in part by a conserved DR5 RARE, is an ancestral feature that was most likely absent in the last common ancestor of all bilaterians and that was thus only subsequently acquired at the base of the deuterostomes.

**Table 1:** Conserved retinoic acid response elements (RAREs) with the characteristic sequence signature AGTTCA(N)_5_AGTTCA identified in the vicinity of bilaterian *CYP26* genes

Species	RARE name	Orientation relative to the CYP26 *gene*	Sequence (5′–3′)	Upstream of	Distance to start codon	Reference
S.p.		+	AGTTCAATAGTTCA	*CYP26*	2503 bp	This study
S.k.		-	AGTTCATACCCAGTTCA	*CYP26a*	137 bp	This study
B.f.	DR5-6	-	AGTTCAACAAAAGTTCA	*CYP26-2*	6405 bp	This study
B.b.	DR5-6	-	AGTTCAACAAAAGTTCA	*CYP26-2*	6740 bp	This study
B.l.	DR5-6	-	AGTTCAACAAAAGTTCA	*CYP26-2*	4083 bp	This study
H.s.	R1	-	AGTTCACCCAAAGTTCA	*CYP26A1*	132 bp	[26]
M.m.	R1	-	AGTTCACCCAAAGTTCA	*CYP26A1*	134 bp	[26]
D.r.	R1	-	AGTTCACACAAAGTTCA	*CYP26A1*	166 bp	[26, 54]
	R2	-	AGTTCAAGGATAGTTCA	*CYP26A1*	1963 bp	
T.r.	R1	-	AGTTCATTCAAAGTTCA	*CYP26A1*	103 bp	This study

Species name abbreviations: B.b. *Branchiostoma belcheri*, B.f. *Branchiostoma floridae*, B.l. *Branchiostoma lanceolatum*, D.r. *Danio rerio*, H.s. *Homo sapiens*, M.m. *Mus musculus*, S.k. *Saccoglossus kowalevskii*, S.p. *Strongylocentrotus purpuratus*, T.r. *Takifugu rubripes*

## Discussion

### *CYP26* genes and their evolutionary involvement in RA-dependent A-P patterning in chordates

**Fig. 9:**
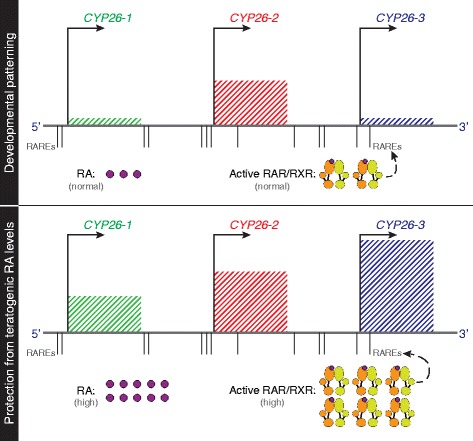
Model for regulation and function of *CYP26* genes in amphioxus. The position of the functionally validated retinoic acid response elements (RAREs) within the amphioxus *CYP26* cluster is indicated relative to the start codons of the three amphioxus *CYP26* genes (*CYP26-1*, *CYP26-2*, and *CYP26-3*). The relative expression of each of the genes is indicated during normal development (normal levels of RA and active RAR/RXR receptor heterodimers) as well as during exposure to RA (high levels of RA and active RAR/RXR receptor heterodimers), hence highlighting the dominance of *CYP26-2* during normal development and the importance of *CYP26-1* and *CYP26-3* for protection against RA teratogenesis

In this study, we assessed the developmental expression patterns and the biological functions of the three *CYP26* duplicates from the European amphioxus *B. lanceolatum*. Globally, our results suggest a sub-functionalization of these genes. Of the three genes, *CYP26-1* and *CYP26-3* are characterized by weak and disperse expression patterns, while *CYP26-2* displays a dynamic, tissue-specific pattern along the A-P axis. Considering that A-P patterning in early stages of amphioxus development is dependent on RA signaling [42, 44], the expression profile of *CYP26-2*, at very distinct positions along the A-P axis of the gastrula and early neurula, is suggestive of a functional role for this gene in this RA-dependent patterning process. This notion is further supported by the results obtained by pharmacological inhibition of CYP26 action during gastrulation, which yielded embryos and larvae that resemble those treated with exogenous RA, displaying severe A-P patterning defects [43]. Thus, we propose that CYP26 enzymes assume two main functions during amphioxus development (Fig. 9), which are equivalent to those observed in vertebrates: (1) the mediation of RA-dependent developmental patterning and (2) the protection against fluctuations of RA levels. Due to its conspicuous and tissue-specific expression during development, we hypothesize that the former is mainly assumed by *CYP26-2*, while the latter is dependent on the activity of *CYP26-1* and *CYP26-3*.

During early development, the expression domains of *CYP26-2* along the A-P axis are inversely correlated with those of *Hox1* and *HNF3-1*, both of which are direct targets of RA signaling in amphioxus [44, 56]. The posterior expression limit of *CYP26-2* in anterior tissues thus abuts the anterior border of both the *Hox1* and *HNF3-1* domains [44]. Concomitantly, the blastopore-associated expression of *CYP26-2* seems to delineate the posterior limits of both *Hox1* and *HNF3-1* [44, 56]. Given that *RAR* expression is ubiquitous during gastrulation [38], the presence of *CYP26-2* in defined domains along the A-P axis might thus be crucial for the creation of RA sinks to subdivide the developing embryo into zones with and without active RA signaling. This is reminiscent of the conserved expression and function of *CYP26A1* during vertebrate gastrulation: *CYP26A1* is expressed in the anterior neural ectoderm and functions to establish A-P boundaries in the developing CNS [18]. It does so by establishing an anterior sink for RA produced posteriorly in the paraxial mesoderm, hence creating a RA gradient along the A-P axis of the neuroectoderm [57, 58].

By the mid neurula stage, the *CYP26-2* signal is present in the entire anterior CNS, with a posterior limit at the boundary between the first and second somite. This *CYP26* expression is hence limited to a region homologous to the vertebrate forebrain and midbrain [59], which is considerably different from what is observed in the vertebrate CNS, where *CYP26* genes are expressed in the developing hindbrain and are fundamental for its patterning along the A-P axis [18, 19]. In the amphioxus hindbrain homolog, RA signaling mediated by RAR/RXR has been shown to confer regional identity along the A-P axis by controlling the collinear expression of *Hox* genes [42]. However, our work suggests that, in contrast to the situation in vertebrates, this process does not involve the deployment of *CYP26* genes in the amphioxus hindbrain homolog. Thus, while the early role for *CYP26* in neural patterning was probably already present in the last common ancestor of amphioxus and vertebrates, a *CYP26*-dependent mechanism for subsequent hindbrain regionalization probably evolved in vertebrates, following the vertebrate-specific *CYP26* duplications.

Following neurulation, the three amphioxus *CYP26* genes are expressed in anterior mesoderm, most noticeably in the anterior-most somites. This is intriguing, as to date there is no convincing evidence for a requirement of RA signaling in amphioxus mesoderm development [41, 42, 60] and the inhibition of CYP26 function by pharmacological treatments does not yield a mesodermal phenotype [43]. These observations raise an important question: why are *CYP26* genes expressed in the anterior amphioxus mesoderm, as they are in vertebrate head mesoderm [25], if they are not required for A-P patterning of this tissue? We speculate that the conspicuous expression of *CYP26* in the anterior somites is an amphioxus innovation and is thus not comparable to the role of *CYP26* in the vertebrate head mesoderm. Instead, the *CYP26* genes might function in the anterior amphioxus mesoderm to establish a buffer zone between the CNS dorsally and the pharynx ventrally, which require distinct RA signaling cues for A-P regionalization [41, 42]. Although further analyses aiming, for example, at the visualization of in vivo RA signaling levels [58] will be required to test this hypothesis, it would nonetheless be interesting to assess, whether this mechanism for separating two gradient-based A-P patterning systems is being used more widely during development of small-sized embryos.

In the course of development, the blastopore-associated expression of amphioxus *CYP26-2* becomes incorporated into the tail bud, a structure that, from the neurula stage on, plays a central role in posterior elongation of the embryo and larva and that is further characterized by the expression of tissue-specific marker genes, such as *Wnt3* [61]. In vertebrates, both *CYP26A1* and *Wnt3a* are also co-expressed in the developing tail bud, and *CYP26A1* null mutants are characterized by a truncated tail and a spatial expression of *Wnt3a* that is abnormally restricted towards the midline of the posterior neural plate [62]. These observations suggest that, in the vertebrate tail bud, *CYP26A1* functions to keep RA levels low to create a permissive environment required for the posterior elongation of the embryo [16, 63]. In contrast, in amphioxus, treatments with CYP26 inhibitor, exogenous RA or the RAR antagonist BMS009 do not affect expression of *Wnt3* in the tail bud [41]. Importantly, these pharmacological treatments, albeit affecting tail fin development, also do not impact posterior elongation of the developing embryo [39, 60]. Together, these findings support the notion that CYP26 function, and more generally the RA signaling system, is not required for tail bud-driven body extension in amphioxus and that this role for RA likely evolved in the vertebrate lineage.

### *CYP26* activity is required for the development of the amphioxus tail fin

Previous studies in the Florida amphioxus, *B. floridae*, have shown that the administration of excess RA during gastrulation results in a small anus [39] and that the continuous administration of RA to *B. floridae* larvae leads to closure of the anus and regression of the tail fin [51]. Our data on the European amphioxus, *B. lanceolatum*, are consistent with these previous findings and further suggest that RA signaling is required for proper tail fin outgrowth. Given that *CYP26-2* is the only *B. lanceolatum CYP26* gene expressed in the posterior ectoderm, it is very likely responsible for fine-tuning endogenous RA signaling levels in this territory.

The amphioxus tail fin is established by columnar epidermal cells that contain a large ciliary rootlet [49, 50] and it has previously been shown that RA promotes the downregulation of a major component of this structure, the protein Rootletin [51]. This downregulation in turn is likely responsible for the induction of tail fin regression upon RA treatments in *B. floridae* larvae [51]. Our results of RA and CYP26 inhibitor treatments in *B. lanceolatum* are generally consistent with these previous observations, although we identified a major difference in the developmental timing of the involvement of RA signaling in tail fin outgrowth between the two amphioxus species. This fact is exemplified by the experimental setups required to obtain tail fin phenotypes. While in *B. floridae* RA-dependent tail fin malformations can only be obtained by continuous treatment of pre-metamorphic larvae with 6–8 gill slits [51], changes in tail fin morphology can be induced much earlier during *B. lanceolatum* development by treating in the course of gastrulation. This suggests that, albeit required for tail fin outgrowth in both amphioxus species, the attenuation of high RA signaling levels in the posterior ectoderm might take place earlier in *B. lanceolatum* than in *B. floridae* development. It is possible that this developmental difference is mediated by a delayed activation of the expression of *CYP26-2* in *B. floridae* relative to *B. lanceolatum*, although additional work is required to support this claim. Altogether, these results indicate that amphioxus tail fin development requires low levels of RA signaling, which are maintained by CYP26 activity. When compared to the known functions of CYP26 in vertebrates, where cells expressing CYP26 enzymes are said to be effectively devoid of RA [63], amphioxus tail fin outgrowth might represent a rare example of a developmental process, where CYP26 is deployed to merely reduce, and not to completely eliminate, RA signaling levels.

### Amphioxus *CYP26* genes are highly responsive RA signaling targets

In vertebrates, it has been established that, in RA-sensitive tissues, RA induces *CYP26* to generate a negative feedback loop that reduces the overall amount of available RA [18]. In mice, for example, expression of both *CYP26A1* and *CYP26B1* is upregulated upon RA treatment [9] and, at least for *CYP26A1*, this regulation is directly mediated by RAR/RXR binding to RAREs in the vicinity of the *CYP26A1* gene [26]. In contrast, the vertebrate *CYP26C1* gene is likely not contributing to this RA-dependent negative feedback system, as its expression is actually downregulated upon RA stimulation [9]. The data presented here suggest that expression of all three amphioxus *CYP26* genes, which are lineage-specific duplicates and located in a single cluster in the genome, is positively regulated by RA signaling. Accordingly, the transcriptional regulation of *B. lanceolatum CYP26-1*, *CYP26-2*, and *CYP26-3* is in all likelihood directly mediated by RAR/RXR heterodimers, given that one DR2-type sequence and 12 DR5-type elements within the *CYP26* cluster are recognized and bound by the *B. lanceolatum* RAR/RXR heterodimer in vitro. Of these 13 in vitro validated RAREs, four DR5 elements are located around the *CYP26-1* coding sequence, four DR5 and one DR2 are found in proximity of *CYP26-2*, and four DR5 elements are associated with the *CYP26-3* gene. Although each of the three amphioxus *CYP26* genes are likely to be directly regulated by RA signaling, the specific arrangement of RAREs relative to the *CYP26* ORFs nonetheless suggests that the expression of each of the three genes is regulated independently by different sets of RAREs. Although this fundamental difference in the regulation of amphioxus and vertebrate *CYP26* genes remains to be demonstrated mechanistically in vivo, it is nonetheless tempting to speculate that alterations in the regulation by RA have been key for redefining the functions of the different *CYP26* genes following their duplication, hence leading to their genomic retention by sub-functionalization.

At least some of the RAREs within the amphioxus *CYP26* cluster might serve as hubs for long-range regulation of gene expression from shared RAREs. Although in both amphioxus and vertebrates it has previously been shown that functional RAREs are generally located in the proximity of genes directly regulated by RA signaling [16, 64], long-range regulatory mechanisms of RA signaling have, for example, been implicated in the control of the rostral expansion of posterior *Hoxb* genes during mouse CNS development [65]. The in vivo validation of the RAREs located within the amphioxus *CYP26* cluster will shed light on their contribution to short- and/or long-range transcriptional control mechanisms exerted by RA signaling. Our *in silico* analyses further revealed the presence, within the amphioxus *CYP26* cluster, of two DR3 elements in close proximity of one of the in vitro validated DR5 elements. Although DR3 elements are generally not recognized by RAR/RXR heterodimers, they are bound by other nuclear receptors, such as vitamin D receptors [66]. The presence of DR3 elements within the amphioxus *CYP26* cluster thus hints at the possibility that additional nuclear receptors are involved in the regulation of amphioxus *CYP26* genes.

Interestingly, the consensus sequence of the in vitro validated amphioxus DR5 RAREs is identical to the classical vertebrate DR5 sequence consensus [(A/G)G(G/T)TCA (N)_5_ (A/G)G(G/T)TCA] [67], suggesting that the DNA binding properties of amphioxus and vertebrate RAR/RXR heterodimers are highly conserved. Along these lines, we found a similar, conserved DR5 RARE in the regulatory region of a hemichordate *CYP26* gene as well as an equivalent DR2 RARE close to the *CYP26* gene of a sea urchin. Whether these elements are recognized by RAR/RXR heterodimers in these two species is currently unknown. Their presence is nevertheless highly suggestive of a biological function [13], which thus indicates that direct regulation of *CYP26* genes by RA signaling is an ancestral feature that was already present in the last common ancestor of all deuterostomes. In contrast, the lack of conserved RAREs in the regulatory regions of the *CYP26* genes of lophotrochozoans, which generally encode *RAR* and *RXR* genes in their genomes [2], support the notion that the direct regulation of *CYP26* transcription by RA signaling is not an ancestral feature of bilaterian animals. This hypothesis is further strengthened by the fact that RARs of gastropod mollusks are unable to bind RA and hence to activate transcription in its presence [68, 69].

### The evolutionary history of *CYP26* genes

In the animal kingdom, the basic molecular components of the RA machinery have previously been described in a wide variety of bilaterian animals, including both protostomes and deuterostomes [2, 45]. Genes encoding the RA degrading enzyme CYP26 have, for example, been identified in the genomes of priapulids, brachiopods, mollusks, annelids, sea urchins, hemichordates, cephalochordates, ascidian tunicates, and vertebrates, but not in those of nematodes and arthropods, suggesting a secondary loss of *CYP26* subfamily genes in these two animal lineages [2, 46]. We also found evidence for possible secondary losses of *CYP26* genes in different vertebrates, including the lamprey *P. marinus* (lacking *CYP26A1*), the chimera *C. milii* (lacking *CYP26A1*), the skate *L. erinacea* (lacking *CYP26C1*), the coelacanth *Latimeria chalumnae* (lacking *CYP26C1*), and the opossum *Monodelphis domestica* (lacking *CYP26B1*) (Additional files 1 and 2). Additional sequence information will be required to validate the absence of these genes from their respective genomes and to assess, at which point in evolution the confirmed gene losses occurred.

Our data further indicate that the evolutionary diversification of the vertebrate *CYP26* genes was a highly complex process. Given the presence of at least one *CYP26A1* and two *CYP26B1/C1* genes in lampreys, the last common ancestor of cyclostomes and gnathostomes probably already possessed at least two *CYP26* genes. Intriguingly, in the lamprey *L. japonicum*, *CYP26A1* and one of the two *CYP26B1/C1*, *CYP26B1/C1a*, are physically linked in a tandem cluster in the genome (on scaffold 19) (Additional file 1), just like *CYP26A1* and *CYP26C1* in gnathostome genomes [70]. This tandem cluster thus very likely originated before the cyclostome-gnathostome split, probably by a tandem duplication event predating the vertebrate-specific WGD. The two linked *CYP26* genes were then duplicated during the first WGD, which took place in the basal vertebrate lineage before the cyclostome-gnathostome split [71–73]. The subsequent loss of one of the duplicated *CYP26A1* genes yielded the *CYP26* complement of extant lampreys, such as *L. japonicum*: one *CYP26A1* and two *CYP26B1/C1* genes. In the gnathostome lineage, additional duplications [71–73] resulted in the diversification of *CYP26B1* and *CYP26C1* genes from the ancestral *CYP26B1/C1*. Although requiring additional scrutiny, this proposed scenario for vertebrate *CYP26* diversification suggests that, if two rounds of WGD occurred in the course of vertebrate evolution, the first likely took place before and the second after the cyclostome-gnathostome split [71, 72]. Alternatively, a single, ancient WGD might have occurred before the cyclostome-gnathostome split and was followed by independent segmental duplications in the cyclostome and gnathostome lineages [73]. Interestingly, in teleost fish, which underwent an additional round of WGD [74], there are also only three *CYP26* genes, one member of each gnathostome paralogy group (*CYP26A1*, *CYP26B1*, and *CYP26C1*), and synteny analyses have revealed that the teleost-specific *CYP26* duplicates have been non-functionalized in the course of evolution, in accordance with the DDC model [30, 31, 70].

Our phylogenetic analyses also provided evidence that *CYP26* genes underwent multiple duplication events, not only in vertebrates, but also in invertebrates, such as cephalochordates, ascidian tunicates, hemichordates, mollusks, annelids, brachiopods, and priapulids. Together, these results suggest that the ancestral bilaterian possessed a single *CYP26* gene that was subjected to independent duplication events in different animal lineages. By correlating the timing of the lineage-specific duplications of invertebrate *CYP26* genes with the geological timescale, we observed that the duplication events fall within three distinct time periods, i.e. the late Ordovician, the early Carboniferous, and the middle Permian/middle Triassic. Thus, the priapulid *CYP26* duplication took place during the late Ordovician, the first cephalochordate duplication and that identified in the hemichordate *S. kowalevskii* in the early Carboniferous, and the second cephalochordate duplication and that in the hemichordate *P.flava* in the middle Permian/middle Triassic. During the late Ordovician, a period marked by a mass extinction of marine species [75], earth was characterized by a rising level of atmospheric O_2_ [76] and by vast shallow and warm continental seas [75]. These conditions were very favorable for the appearance of cyanobacterial mats at moderate depths of the water column [77, 78]. Similarly, during the early Carboniferous atmospheric O_2_ levels were rising [76, 79], and the fossil record accounts for one of the highest concentrations of calcified marine cyanobacteria [78]. The middle Permian/middle Triassic, in turn, saw the greatest biotic crisis in earth’s history [80], and the fossil record suggests that, as a result of environmental changes, cyanobacteria became one of the most abundant life forms in both shallow and deep water environments [81].

Extant cyanobacteria that are known to create massive blooms under the exact same environmental conditions include *Trichodesmium*, *Anabaena*, and *Synechocystis* [82, 83], all of which are also known to produce high levels of carotenoids and retinoids as anti-oxidative byproducts, which have been shown to induce teratogenic effects in the surrounding fauna [83–85]. The prevalence of cyanobacterial mats in marine environments during the late Ordovician, early Carboniferous, and the middle Permian/middle Triassic might thus explain why *CYP26* duplications that occurred during these two geological periods were independently retained, by either neo- or sub-functionalization [30, 31], in the genomes of different marine animal lineages. The independent duplication of *CYP26* genes might have increased the overall fitness of a given population by favoring individuals that were more efficient in buffering fluctuations of exogenous retinoid levels. Altogether, this finding represents an intriguing example of adaptive convergent evolution in response to environmental changes.

## Conclusions

In the present study, we characterized the expression, function, and regulation of *CYP26* genes during amphioxus development. Our data suggest that, despite the independent origins of the *CYP26* gene repertoires in chordates, the *CYP26* genes of cephalochordates and vertebrates convergently evolved similar developmental functions: RA-dependent patterning and homeostatic regulation of RA levels. Moreover, by comparing the regulatory regions of *CYP26* genes in three amphioxus species with those from several different animal taxa, we identified a highly conserved, functional RARE, suggesting that negative feedback regulation of RA signaling is an evolutionary ancient mechanism for controlling endogenous RA levels. This mechanism of regulation was likely already present in the last common ancestor of all deuterostomes, but not in that of all bilaterians. Finally, the correlation between the timing of lineage-specific duplications of bilaterian *CYP26* genes and major environmental changes in the geological record suggest that the evolutionary diversification of the *CYP26* subfamily in bilaterians was strongly influenced by environmental pressures to buffer fluctuations of exogenous retinoid levels. In sum, this work thus sheds light on the evolution of the regulation of endogenous RA levels and establishes a framework for studying adaptive convergent evolutionary changes following gene duplication.

## Methods

### Amphioxus adult husbandry, embryo rearing, and pharmacological treatments

Sexually mature animals of the European amphioxus (*Branchiostoma lanceolatum*) were collected by dredging in Argelès-sur-Mer, France, and retrieved from the sand by sieving. The collected animals were split evenly into tanks, with about 10–15 animals per aquarium, males and females together. The water temperature in the aquaria was kept at 16–17 °C, and the animals were kept under a spring-like day/night period, with 14 h of light and 10 h of absolute darkness. Spawning was induced by a 36-h thermal shock at 23 °C, as previously described [86–88]. Following oocyte and sperm collection and in vitro fertilization, the embryos were raised in artificial seawater, in the dark, at 19 °C [89]. Pharmacological treatments of *B. lanceolatum* embryos were performed at the late blastula stage (6 h of development) with all-*trans* RA (at 0,1 μM and 1 μM) (Sigma-Aldrich, Saint-Quentin Fallavier, France), the RAR antagonists BMS009 (at 1 μM) or BMS493 (at 1 μM) (Sigma-Aldrich, Saint-Quentin Fallavier, France), the CYP26 inhibitor R115866 (at 0,5 μM and 0,1 μM) (provided by Janssen Research & Development, a division of Janssen Pharmaceutica NV, Beerse, Belgium) or with a combination of both BMS493 (at 1 μM) and R115866 (at 0,5 μM). All compounds were initially dissolved in dimethyl sulfoxide (DMSO) to create 1000X stock solutions and subsequently added to the embryo cultures in artificial seawater in a 1:1000 dilution to yield the respective final concentrations. As a control, embryos were treated in separate dishes with DMSO alone to a final dilution of 1:1000 [38, 39]. Puromycin (Sigma-Aldrich, Saint-Quentin Fallavier, France) treatments were performed at the mid neurula stage (19 h of development) by adding the compound to embryo cultures at a final concentration of 200 μg/ml. After 5 min of incubation, all-*trans* RA (1 μM), the RAR antagonist BMS009 (1 μM) or DMSO (at a dilution of 1:1000) were added, and, 1 h thereafter, the embryos were frozen for RNA extraction.

### Sequence analyses and gene cloning

The *B. lanceolatum CYP26-1, CYP26-2*, and *CYP26-3* sequences were obtained *in silico* from the *B. lanceolatum* genome by local BLAST using as template the sequences of *B. floridae* and *B. belcheri CYP26* (Additional file 1). RNA was extracted from embryos at different developmental stages according to the established protocols [90] and cDNA was synthesized using the SuperScriptIII reverse transcription kit (Invitrogen, Cergy Pontoise, France). Complete coding sequences of *B. lanceolatum CYP26-1, CYP26-2*, and *CYP26-3* were subsequently cloned by PCR using gene-specific primers containing the start and stop codons of the three *CYP26* genes. RACE-PCR experiments using the SMARTer™ RACE cDNA amplification kit (Clontech, Saint-Germain-en-Laye, France) were then performed to amplify the 5′ and 3′ untranslated regions (UTRs) of *B. lanceolatum CYP26-1, CYP26-2*, and *CYP26-3*. The PCR and RACE-PCR products were cloned into the pCRII-TOPO vector (Invitrogen, Cergy Pontoise, France) and sequenced on both strands for validation. The *B. lanceolatum CYP26-1, CYP26-2*, and *CYP26-3* sequences were deposited in GenBank and their accession numbers are as follows: *CYP26-1* (KX118106), *CYP26-2* (KX118108), and *CYP26-3* (KX118107). Furthermore, a 2291-bp piece of the *B. lanceolatum Rootletin* gene was amplified by PCR, cloned into the pGEM-T Easy vector (Promega, Charbonnières-les-Bains, France), and verified by sequencing on both strands (GenBank accession number: KX118111), before being used as a marker for in situ hybridization (ISH) experiments.

### Phylogenetic analyses

Phylogenetic trees of the *CYP26* subfamily were calculated from amino acid and nucleotide sequences and the sequences included in the analysis are listed in Additional file 1, along with representative members of the *CYP51* subfamily, which were used as outgroup. Nucleotide sequences were translated into amino acid sequences, aligned with Muscle as implemented in SeaView v4.5.4 [91], and refined by eye (Additional file 5). The final amino acid alignment was subsequently retransformed into the final nucleotide alignment (Additional file 6). The best-fit models of amino acid and nucleotide sequence evolution were selected based on the AIC score implemented, respectively, in ProtTtest 3 [92] and jModelTest v2.1 [93]. Molecular phylogenies were calculated with the Maximum Likelihood (ML) method using PhyML v3.1 [94] under the models selected by ProtTtest 3 (LG + I + G + F) and jModelTest v2.1 (GTR + I + G). In addition, Bayesian Inference (BI) analyses were performed using the program MrBayes v3.2.6 [95] and the same models. The robustness of each node was estimated by bootstrap analyses (in 1000 pseudoreplicates) using PhyML v3.1 for the ML tree and by posterior probability for the BI tree, with two Monte Carlo Markov Chain (MCMC) analyses run independently for 100 million generations and trees sampled every 1000 generations. The burn-in was determined with Tracer v1.6 [96], and the average standard deviation of split frequencies remained at <0.05 after the burn-in threshold. 10% and 50% of the trees were discarded, respectively, for the amino acid and nucleotide datasets. Consensus trees were visualized with Figtree v1.4 [97].

### Comparative dating analysis

Based on the nucleotide dataset, the divergence dates of the *CYP26* sequences were estimated with Beast v2.4 [98] using divergence times in million years ago (Mya) estimated with the calibration intervals proposed by Benton and colleagues and dos Reis and colleagues [99, 100]: origin of Vertebrata (457,5-636,1 Mya), Euarchontoglires (61,6-164,6 Mya), and Hemichordata (504,5-636,1 Mya) as well as the divergence between Holostei and Teleostei (250,0–331,1 Mya) and between Otocephala and Euteleostei (150,94–235 Mya). All calibration constraint sets were defined following a hard minimum, soft upper boundaries, and a lognormal prior [98]. Monte Carlo Markov Chain (MCMC) analyses were run on the nucleotide dataset for 100 million generations with trees sampled every 1000 generations [98]. Convergence of the calculations was verified and burn-in estimated with Tracer v1.6 [96]. The results of the MCMC run were sampled with LogCombiner and a burn-in of 30% [98]. The trees were combined into a maximum clade credibility tree using TreeAnnotator with an estimation of the mean node height and highest posterior density intervals fixed at 95% [98].

### *B. lanceolatum CYP26* cluster reconstitution and identification of putative RAREs

The full-length ORFs of *B. lanceolatum CYP26-1*, *CYP26-2*, and *CYP26-3* were used as templates for local BLAST searches of the *B. lanceolatum* genome to identify scaffolds containing UTRs and coding regions of *CYP26-1*, *CYP26-2*, and *CYP26-3*. Sequences of genome regions not obtained by these BLAST approaches (hence corresponding to gaps in the *B. lanceolatum CYP26* cluster) were subsequently identified by reciprocal BLAST searches of the *B. lanceolatum* genome sequence using short regions (less than 2 Kbp) of the *B. floridae* and *B. belcheri CYP26* clusters. Putative RARE sequences were identified with an automated pipeline [51] using as input RAREs previously described as functional in amphioxus as well as all possible RARE combinations resulting from the canonical vertebrate RARE consensus: (A/G)G(G/T)TCA(N)_0–9_(A/G)G(G/T)TCA. Detailed sequence and scaffold information is provided in Additional files 4 and 7.

### Multiple expectation maximization algorithm for motif elicitation (MEME) analysis

Multiple expectation maximization algorithm for motif elicitation (MEME) logos were calculated with MEME Suite v4.10.1 [101] using as input file the sequences of the in vitro validated *B. lanceolatum* DR5 RARE sequences including 5 nucleotides upstream and downstream of the element. The following settings were used to obtain the MEME logos: nmotifs = 2, minwidth = 15, maxwidth = 27.

### Electrophoretic mobility shift assay (EMSA) experiments

The *B. lanceolatum RAR* and *RXR* coding sequences were amplified by PCR and cloned into the pGEM-T Easy vector (Promega, Charbonnières-les-Bains, France). Subcloning into the pCS2+ vector [102] was performed using introduced EcoRI and XhoI restriction sites. Following verification by sequencing on both strands of the RAR-pCS2+ and RXR-pCS2+ constructs (GenBank accession numbers: *B. lanceolatum RAR*, KX118109; *B. lanceolatum RXR*, KX118110), RAR and RXR proteins were produced by in vitro translation using the TNT coupled reticulocyte lysate system (Promega, Charbonnières-les-Bains, France). Electrophoretic mobility shift assays (EMSAs) were performed as previously described [69] using 4 μl of each receptor synthesized in vitro and 30–50 × 10^3^ CPM of double-stranded oligonucleotide probe end-labeled with (γ-^32^P)ATP, incubated for 30 min on ice in a final volume of 20 μl of binding buffer: 20 mM Tris–HCl pH8, 50 mM KCl, 2 mM DTT, 25 mM MgCl_2_, 50 mM NaCl, 1 μg poly (dI-dC), and 10% glycerol. The samples were subsequently run on a 5% native acrylamide gel in 1X TAE for 2 h.

### In situ hybridization (ISH), histology, imaging, and tail fin measurements

Antisense riboprobe synthesis, ISH, and Hoechst staining (Invitrogen, Cergy Pontoise, France) experiments were performed as previously described [103]. For ISH, Hoechst staining, and tail fin measurements, amphioxus (*B. lanceolatum*) embryos and larvae were fixed in 4% paraformaldehyde at different developmental stages, as previously described [103]. Following ISH, *B. lanceolatum* embryos and larvae were first photographed as whole mounts using Zeiss DIC (differential interference contrast) optics (Carl Zeiss SAS, Marly le Roi, France) and subsequently counterstained in Ponceau S (Sigma-Aldrich, Saint-Quentin Fallavier, France), embedded in Spurr’s resin (Sigma-Aldrich, Saint-Quentin Fallavier, France), and prepared as 3 μm sections for light microscopy observations and photography. Following Hoechst staining, *B. lanceolatum* larvae were embedded in Mowiol mounting medium (Sigma-Aldrich, Saint-Quentin Fallavier, France) overnight at 4 °C, before being imaged using a Leica TCS SP5 confocal microscope (Leica Microsystems SAS, Nanterre, France). ImageJ was subsequently used for image processing and for the creation of maximal projections [104]. For tail fin measurements, normal and treated *B. lanceolatum* larvae were photographed using Zeiss DIC optics (Carl Zeiss SAS, Marly le Roi, France). The length of the tail fin, defined as the distance between the anterior-most end of the tail fin ectoderm and the posterior-most tip of the tail fin (as represented in Fig. 3g), was subsequently measured using the measurement tool of ImageJ [104] and ultimately represented as mean ± standard deviation, with *n* = 15 for each treatment and control conditions. Student’s t-test was used to assess the statistical significance of the length differences measured in the treated specimens relative to the DMSO control.

### Quantitative real time PCR (qPCR) assays

Quantitative real time PCR (qPCR) experiments were performed at two developmental stages (mid neurula at 20 h of development and early larva at 48 h of development). In addition to the puromycin-treated material described above, the cDNAs from DMSO treatment controls as well as from embryos treated at the late blastula stage (6 h of development) with either 1 μM all-*trans* RA or 1 μM of the RAR antagonist BMS009 were assayed on a MJ Research DNA Engine Opticon system (Bio-Rad, Marnes-la-Coquette, France) using the QuantiTect SYBR Green PCR reagent (Qiagen SAS, Courtaboeuf, France) and primers specific for *B. lanceolatum CYP26-1*, *CYP26-2*, *CYP26-3*, *RAR*, and *18S *rRNA (Additional file 8). Based on the lack of response to the different pharmacological treatments assayed, *18S *rRNA was selected as the reference for internal standardization of the starting quantity of RNA. Each qPCR experiment was performed in triplicates and the relative expression was normalized to *RAR* or *CYP26-2* expression levels, in Figs. 4 and 6, respectively. Normalized expression levels are shown as ΔΔCT means ± standard deviation, with n = 3. Furthermore, fold change of expression relative to the control is represented as the mean of the ΔΔCT ratios of all possible combinations of the three replicates of a given condition over the three controls ± standard deviation, with n = 9.

## Additional files

Additional file 1: Accession numbers of the *CYP26* and *CYP51* subfamily sequences used to calculate the phylogenetic trees and to carry out the dating analyses. Species names and database information are provided for each sequence. (XLSX 12 kb)
Additional file 2: Phylogenetic tree of the *CYP26* subfamily, with *CYP51* as outgroup. Tree calculated with the Bayesian Inference (BI) and Maximum Likelihood (ML) methods based on 15 vertebrate species (42 *CYP26* sequences), 1 tunicate species (2 *CYP26* sequences), 3 cephalochordate species (9 *CYP26* sequences), 3 ambulacrarian species (6 *CYP26* sequences), 5 lophotrochozoan species (12 *CYP26* sequences), and 1 ecdysozoan species (2 *CYP26* sequences) (for details see Additional file 1). The robustness of each node was assessed by posterior probability (for the BI phylogeny) and bootstrap (for the ML phylogeny) analyses, which are indicated at each node: posterior probabilities (ranging from 0 to 1)/bootstrap percentages (ranging from 0 to 100). “--” indicates nodes where the ML tree did not recover the branching pattern of the BI tree or nodes with bootstrap support inferior to 50%. Branch lengths are representative of the amino acid substitution rate. Species name abbreviations: B.b., *Branchiostoma belcheri* (in blue); B.f., *Branchiostoma floridae* (in red); B.l., *Branchiostoma lanceolatum* (in green); C.g., *Crassostrea gigas*; C.i., *Ciona intestinalis*; C.m., *Callorhinchus milii;* C.t., *Capitella teleta*; D.r., *Danio rerio*; G.a., *Gasterosteus aculeatus*; G.g., *Gallus gallus*; H.s., *Homo sapiens*; L.a. *Lingula anatina*; L.c., *Latimeria chalumnae*; L.e., *Leucoraja erinacea*; L.g., *Lottia gigantea*; L.j., *Lethenteron japonicum*; L.o., *Lepisosteus oculatus*; M.d., *Monodelphis domestica*; M.m., *Mus musculus*; O.b., *Octopus bimaculoides*; O.l., *Oryzias latipes*; P.c., *Priapulus caudatus*; P.f., *Ptychodera flava*; P.m., *Petromyzon marinus*; S.k., *Saccoglossus kowalevskii*; S.p., *Strongylocentrotus purpuratus*; T.r., *Takifugu rubripes*; X.t., *Xenopus tropicalis*. (PDF 559 kb)
Additional file 3: Comparative dating tree for the *CYP26* subfamily. Divergence dates of the *CYP26* sequences were estimated using fossil-based calibration intervals. Cladogram node dates represent estimation of the mean node height and the highest posterior density intervals fixed at 95% are shown in parentheses. Geological era abbreviations: Cam, Cambrian; Car, Carboniferous; Cre, Cretaceous; Dev, Devonian; Jur, Jurassic; Ord, Ordovician; Per, Permian; Sil, Silurian; Tri, Triassic. Species name abbreviations: B.b., *Branchiostoma belcheri* (in blue); B.f., *Branchiostoma floridae* (in red); B.l., *Branchiostoma lanceolatum* (in green); C.g., *Crassostrea gigas*; C.i., *Ciona intestinalis*; C.m., *Callorhinchus milii;* C.t., *Capitella teleta*; D.r., *Danio rerio*; G.a., *Gasterosteus aculeatus*; G.g., *Gallus gallus*; H.s., *Homo sapiens*; L.a. *Lingula anatina*; L.c., *Latimeria chalumnae*; L.e., *Leucoraja erinacea*; L.g., *Lottia gigantea*; L.j., *Lethenteron japonicum*; L.o., *Lepisosteus oculatus*; M.d., *Monodelphis domestica*; M.m., *Mus musculus*; O.b., *Octopus bimaculoides*; O.l., *Oryzias latipes*; P.c., *Priapulus caudatus*; P.f., *Ptychodera flava*; P.m., *Petromyzon marinus*; S.k., *Saccoglossus kowalevskii*; S.p., *Strongylocentrotus purpuratus*; T.r., *Takifugu rubripes*; X.t., *Xenopus tropicalis*. (PDF 271 kb)
Additional file 4: Conserved retinoic acid response elements (RAREs) found in the *Branchiostoma lanceolatum*, *Branchiostoma belcheri*, and *Branchiostoma floridae CYP26* clusters. *B. lanceolatum* RAREs recognized and bound by the *B. lanceolatum* RAR/RXR heterodimer in vitro (Fig. 8) are indicated in black, RAREs that do not associate with the *B. lanceolatum* RAR/RXR heterodimer in vitro are shown in grey. The in vitro validated *B. lanceolatum* RAREs composed of two direct repeats (DRs) and separated by a 5 nucleotide spacer (i.e. the validated *B. lanceolatum* DR5-type RAREs) were used to calculate a consensus *B. lanceolatum* DR5 RARE by multiple expectation maximization algorithm for motif elicitation (MEME) analysis. (XLSX 77 kb)
Additional file 5: Amino acid alignment used for phylogenetic tree reconstruction analyses of the CYP26 subfamily. (NEX 46 kb)
Additional file 6: DNA alignment used for comparative dating analyses of the *CYP26* subfamily. (NEX 135 kb)
Additional file 7: Species names, corresponding genome access, and scaffold information related to the sequences used as input for the *in silico* identification of conserved putative retinoic acid response elements (RAREs). (XLSX 9 kb)
Additional file 8: Sequences of the oligonucleotides used as primers for quantitative real time PCR (qPCR) analyses of the indicated *Branchiostoma lanceolatum* genes. (XLSX 8 kb)

## Abbreviations

A-P: Anterior-posterior
BI: Bayesian inference
BLAST: Basic local alignment search tool
BMS: Bristol-Myers Squibb
cDNA: Complimentary deoxyribonucleic acid
CNS: Central nervous system
CYP26: Cytochrome P450 subfamily 26
CYP51: Cytochrome P450 subfamily 51
DDC: Duplication degeneration complementation
DIC: Differential interference contrast
DMSO: Dimethyl sulfoxide
DR: Direct repeat
EMSA: Electrophoretic mobility shift assay
FGF: Fibroblast growth factor
HNF: Hepatocyte nuclear factor
Hox: Homeobox gene
ISH: In situ hybridization
Kbp: Kilo base pairs = 1000 base pairs
MCMC: Monte Carlo Markov chain
MEME: Multiple expectation maximization algorithm for motif elicitation
ML: Maximum likelihood
Mya: Million years ago
ORF: Open reading frame
qPCR: Quantitative real time polymerase chain reaction
RA: Retinoic acid
RACE: Rapid amplification of complimentary deoxyribonucleic acid ends
RALDH: Retinaldehyde dehydrogenase
RAR: Retinoic acid receptor
RARE: Retinoic acid response element
rRNA: Ribosomal ribonucleic acid
RXR: Retinoid X receptor
UTR: Untranslated region
WGD: Whole genome duplication
Wnt: Wingless-related integration site
ΔΔCT: Delta delta threshold cycle
