# Supplementary figures and images for: Lineage-specific duplication of amphioxus retinoic acid degrading enzymes (CYP26) resulted in sub-functionalization of patterning and homeostatic roles
Source: BMC Evol Biol. 2017 Jan 19;17:24. doi: 10.1186/s12862-016-0863-1 (PMC5247814; doi:10.1186/s12862-016-0863-1)

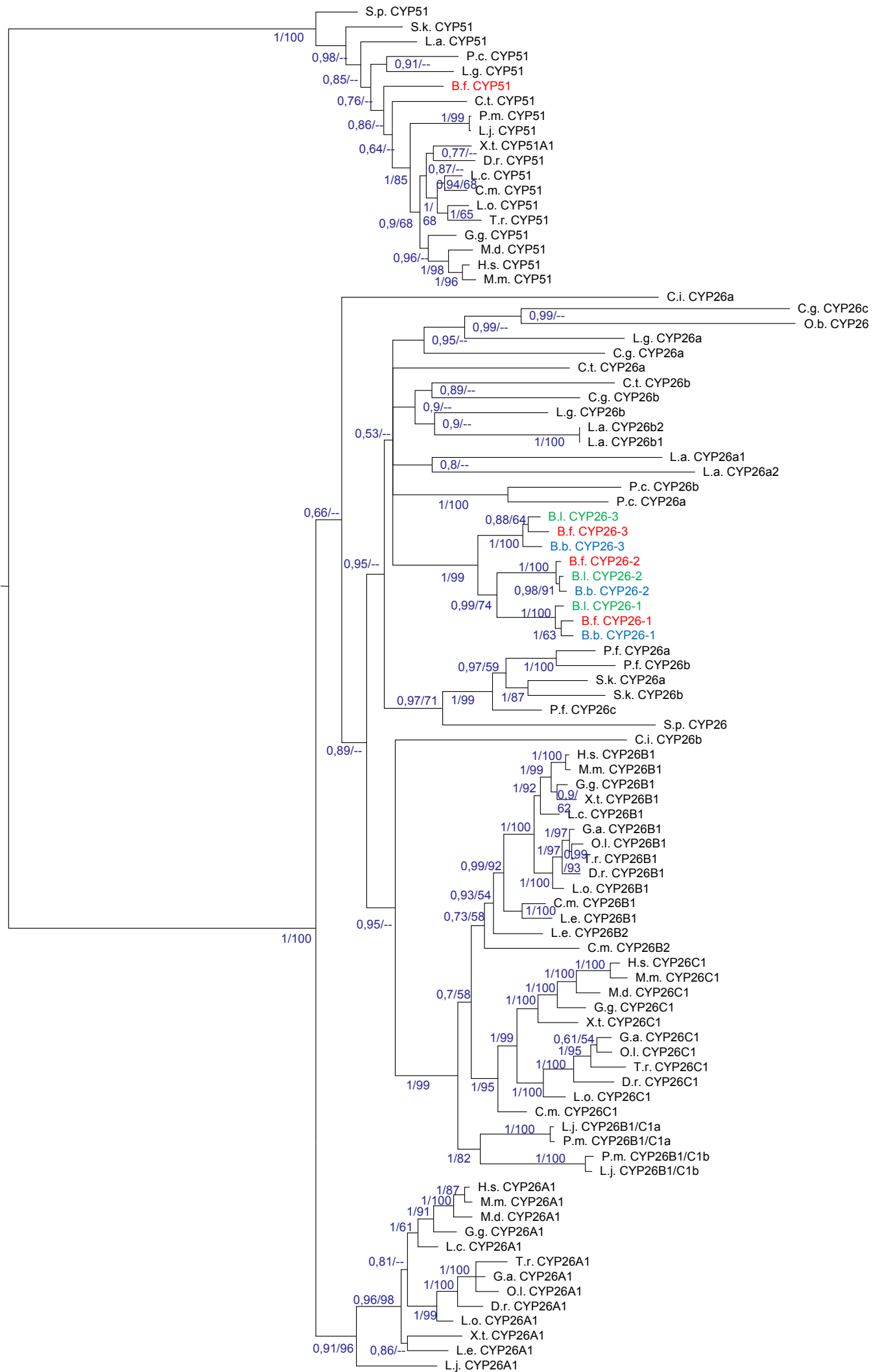

Supplement: Additional file 2: — Phylogenetic tree of the CYP26 subfamily, with CYP51 as outgroup. Tree calculated with the Bayesian Inference (BI) and Maximum Likelihood (ML) methods based on 15 vertebrate species (42 CYP26 sequences), 1 tunicate species (2 CYP26 sequences), 3 cephalochordate species (9 CYP26 sequences), 3 ambulacrarian species (6 CYP26 sequences), 5 lophotrochozoan species (12 CYP26 sequences), and 1 ecdysozoan species (2 CYP26 sequences) (for details see Additional file 1). The robustness of each node was assessed by posterior probability (for the BI phylogeny) and bootstrap (for the ML phylogeny) analyses, which are indicated at each node: posterior probabilities (ranging from 0 to 1)/bootstrap percentages (ranging from 0 to 100). “--” indicates nodes where the ML tree did not recover the branching pattern of the BI tree or nodes with bootstrap support inferior to 50%. Branch lengths are representative of the amino acid substitution rate. Species name abbreviations: B.b., Branchiostoma belcheri (in blue); B.f., Branchiostoma floridae (in red); B.l., Branchiostoma lanceolatum (in green); C.g., Crassostrea gigas; C.i., Ciona intestinalis; C.m., Callorhinchus milii; C.t., Capitella teleta; D.r., Danio rerio; G.a., Gasterosteus aculeatus; G.g., Gallus gallus; H.s., Homo sapiens; L.a. Lingula anatina; L.c., Latimeria chalumnae; L.e., Leucoraja erinacea; L.g., Lottia gigantea; L.j., Lethenteron japonicum; L.o., Lepisosteus oculatus; M.d., Monodelphis domestica; M.m., Mus musculus; O.b., Octopus bimaculoides; O.l., Oryzias latipes; P.c., Priapulus caudatus; P.f., Ptychodera flava; P.m., Petromyzon marinus; S.k., Saccoglossus kowalevskii; S.p., Strongylocentrotus purpuratus; T.r., Takifugu rubripes; X.t., Xenopus tropicalis. (PDF 559 kb) [file 12862_2016_863_MOESM2_ESM.pdf]

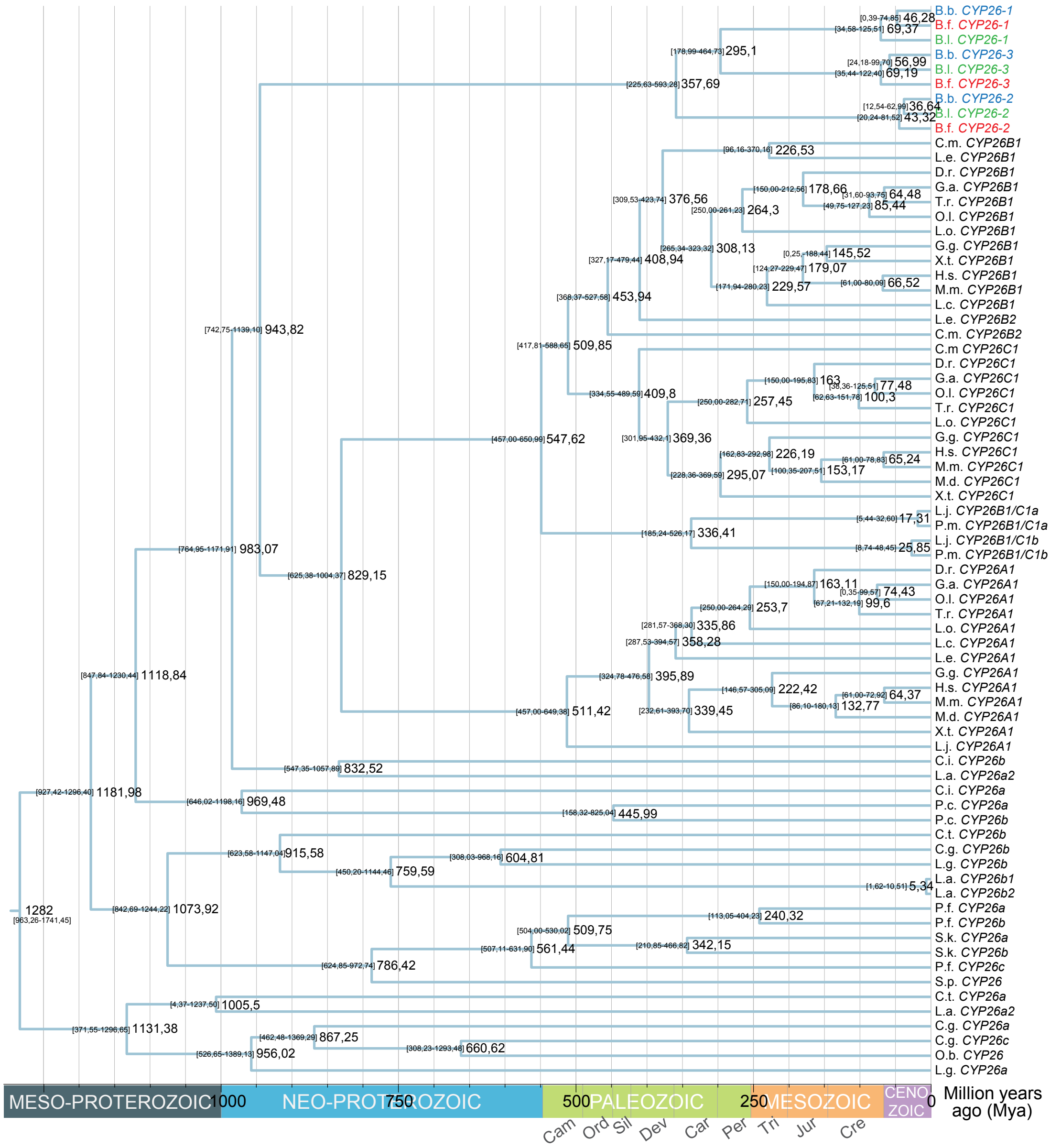

Supplement: Additional file 3: — Comparative dating tree for the CYP26 subfamily. Divergence dates of the CYP26 sequences were estimated using fossil-based calibration intervals. Cladogram node dates represent estimation of the mean node height and the highest posterior density intervals fixed at 95% are shown in parentheses. Geological era abbreviations: Cam, Cambrian; Car, Carboniferous; Cre, Cretaceous; Dev, Devonian; Jur, Jurassic; Ord, Ordovician; Per, Permian; Sil, Silurian; Tri, Triassic. Species name abbreviations: B.b., Branchiostoma belcheri (in blue); B.f., Branchiostoma floridae (in red); B.l., Branchiostoma lanceolatum (in green); C.g., Crassostrea gigas; C.i., Ciona intestinalis; C.m., Callorhinchus milii; C.t., Capitella teleta; D.r., Danio rerio; G.a., Gasterosteus aculeatus; G.g., Gallus gallus; H.s., Homo sapiens; L.a. Lingula anatina; L.c., Latimeria chalumnae; L.e., Leucoraja erinacea; L.g., Lottia gigantea; L.j., Lethenteron japonicum; L.o., Lepisosteus oculatus; M.d., Monodelphis domestica; M.m., Mus musculus; O.b., Octopus bimaculoides; O.l., Oryzias latipes; P.c., Priapulus caudatus; P.f., Ptychodera flava; P.m., Petromyzon marinus; S.k., Saccoglossus kowalevskii; S.p., Strongylocentrotus purpuratus; T.r., Takifugu rubripes; X.t., Xenopus tropicalis. (PDF 271 kb) [file 12862_2016_863_MOESM3_ESM.pdf]
